# Physiology and effects of nucleosides in mice lacking all four adenosine receptors

**DOI:** 10.1371/journal.pbio.3000161

**Published:** 2019-03-01

**Authors:** Cuiying Xiao, Naili Liu, Kenneth A. Jacobson, Oksana Gavrilova, Marc L. Reitman

**Affiliations:** 1 Diabetes, Endocrinology, and Obesity Branch, National Institute of Diabetes and Digestive and Kidney Diseases, NIH, Bethesda, Maryland, United States of America; 2 Mouse Metabolism Core, National Institute of Diabetes and Digestive and Kidney Diseases, NIH, Bethesda, Maryland, United States of America; 3 Molecular Recognition Section, Laboratory of Bioorganic Chemistry, National Institute of Diabetes and Digestive and Kidney Diseases, NIH, Bethesda, Maryland, United States of America; Columbia University, UNITED STATES

## Abstract

Adenosine is a constituent of many molecules of life; increased free extracellular adenosine indicates cell damage or metabolic stress. The importance of adenosine signaling in basal physiology, as opposed to adaptive responses to danger/damage situations, is unclear. We generated mice lacking all four adenosine receptors (ARs), *Adora1*^*−/−*^*;Adora2a*^*−/−*^*;Adora2b*^*−/−*^*;Adora3*^*−/−*^ (quad knockout [QKO]), to enable investigation of the AR dependence of physiologic processes, focusing on body temperature. The QKO mice demonstrate that ARs are not required for growth, metabolism, breeding, and body temperature regulation (diurnal variation, response to stress, and torpor). However, the mice showed decreased survival starting at about 15 weeks of age. While adenosine agonists cause profound hypothermia via each AR, adenosine did not cause hypothermia (or bradycardia or hypotension) in QKO mice, indicating that AR-independent signals do not contribute to adenosine-induced hypothermia. The hypothermia elicited by adenosine kinase inhibition (with A134974), inosine, or uridine also required ARs, as each was abolished in the QKO mice. The proposed mechanism for uridine-induced hypothermia is inhibition of adenosine transport by uridine, increasing local extracellular adenosine levels. In contrast, adenosine 5′-monophosphate (AMP)–induced hypothermia was attenuated in QKO mice, demonstrating roles for both AR-dependent and AR-independent mechanisms in this process. The physiology of the QKO mice appears to be the sum of the individual knockout mice, without clear evidence for synergy, indicating that the actions of the four ARs are generally complementary. The phenotype of the QKO mice suggests that, while extracellular adenosine is a signal of stress, damage, and/or danger, it is less important for baseline regulation of body temperature.

## Abstract 2

A study of mice lacking all four adenosine receptors shows that while they mediate effects of uridine, inosine and adenosine, these receptors are dispensable for growth, metabolism, breeding, and body temperature regulation. This suggests that extracellular adenosine is a damage or danger signal, rather than a major regulator of baseline physiology.

## Author summary

Elevated extracellular adenosine generally indicates metabolic stress or cell damage and regulates many aspects of physiology. We studied “QKO” mice lacking all four adenosine receptors. Young QKO mice do not appear obviously ill, but do show decreased survival later in life. QKO mice demonstrate that adenosine receptors are not required for growth, metabolism, breeding, and body temperature regulation. QKO mice are missing the pharmacologic effects of adenosine on body temperature, heart rate, and blood pressure. Therefore, all of these effects are mediated by the four adenosine receptors. We also determined that the hypothermic effects of a pharmacologic adenosine kinase inhibitor (A134974), uridine, or inosine each requires adenosine receptors. The uridine-induced hypothermia is likely due to its inhibition of adenosine uptake into cells. QKO mouse physiology appears to be the sum of the individual knockout mice, without evidence for synergy, indicating that the actions of the four adenosine receptors are generally complementary.

## Introduction

The nucleoside adenosine is incorporated covalently into many molecules of life, including those involved in information archiving and translation, energy storage and use, regulation of gene expression and function, and intermediary metabolism. Free, extracellular adenosine levels are normally low, but are increased by cell damage and metabolic stressors, such as hypoxia and seizures. Elevated extracellular adenosine thus indicates danger or damage to tissues and elicits adaptive responses. These responses are typically adaptations to the danger state, including selective regulation of perfusion and dampening of (presumably excessive) inflammatory/immune responses. A central question in adenosine physiology is the contribution of adenosine to basal homeostasis versus its clear role in stressed states [1,2].

Adenosine signals via four G protein–coupled adenosine receptors (ARs), A_1_AR, A_2A_AR, A_2B_AR, and A_3_AR. A_1_AR and A_3_AR are generally coupled to Gi, reducing cAMP and considered inhibitory, while A_2A_AR and A_2B_AR are typically coupled to Gs/Gq and are stimulatory, increasing cAMP/Ca^++^ [3]. AR activation causes a plethora of physiology, with some actions unique to one AR and others with contributions from multiple ARs.

Approved AR drugs include adenosine for paroxysmal supraventricular tachycardia (A_1_AR agonism), adenosine and regadenoson for myocardial perfusion imaging (A_2A_AR agonism, causing coronary artery vasodilation [4]), istradefylline for Parkinson’s disease (A_2A_AR antagonism [5]), and the nonselective AR antagonist theophylline for asthma. Caffeine, another nonselective AR antagonist, is found in coffee and certain medications for treatment of drowsiness, headache, or migraines. Selective AR ligands are being investigated for additional indications [6,7]. Other drugs, such as dipyridamole and low-dose methotrexate, increase local adenosine levels, thus secondarily activating ARs.

One physiologic effect of adenosine is hypothermia [8]. Adenosine-induced hypothermia was initially attributed to brain A_1_AR [9,10], possibly through ARs in the nucleus of the solitary tract [11] or preoptic area [12,13]. A_1_AR may also regulate torpor (a hibernation-like state with hypothermia and reduced metabolism, heart rate, and respiration) [14], but neither A_1_AR nor A_3_AR is required for fasting-induced torpor [15]. Additionally, activation of mouse mast cell A_3_ARs also causes hypothermia [16–18], via histamine release and signaling through histamine H_1_ receptors [19,20]. Hypothermia is also caused by A_2A_AR agonists, which produce vasodilation, and by A_2B_AR agonists, which may be acting centrally [21]. Thus, agonism of any of the four ARs can produce hypothermia.

It is unknown if adenosine also causes hypothermia by additional mechanisms. Could cellular uptake and phosphorylation of adenosine increase adenosine 5′-monophosphate (AMP)/ATP ratios, activating the energy sensor adenosine monophosphate–activated protein kinase (AMPK) [22]? AMPK is proposed to have a role in signaling torpor/hypothermia [23]. We produced *Adora1*^*−/−*^*;Adora2a*^*−/−*^*;Adora2b*^*−/−*^*;Adora3*^*−/−*^ mice (quad knockout [QKO] mice) to examine this idea and study the basal physiology and pathophysiology of mice lacking all four ARs.

## Results

### Producing QKO mice

**Fig 1:**
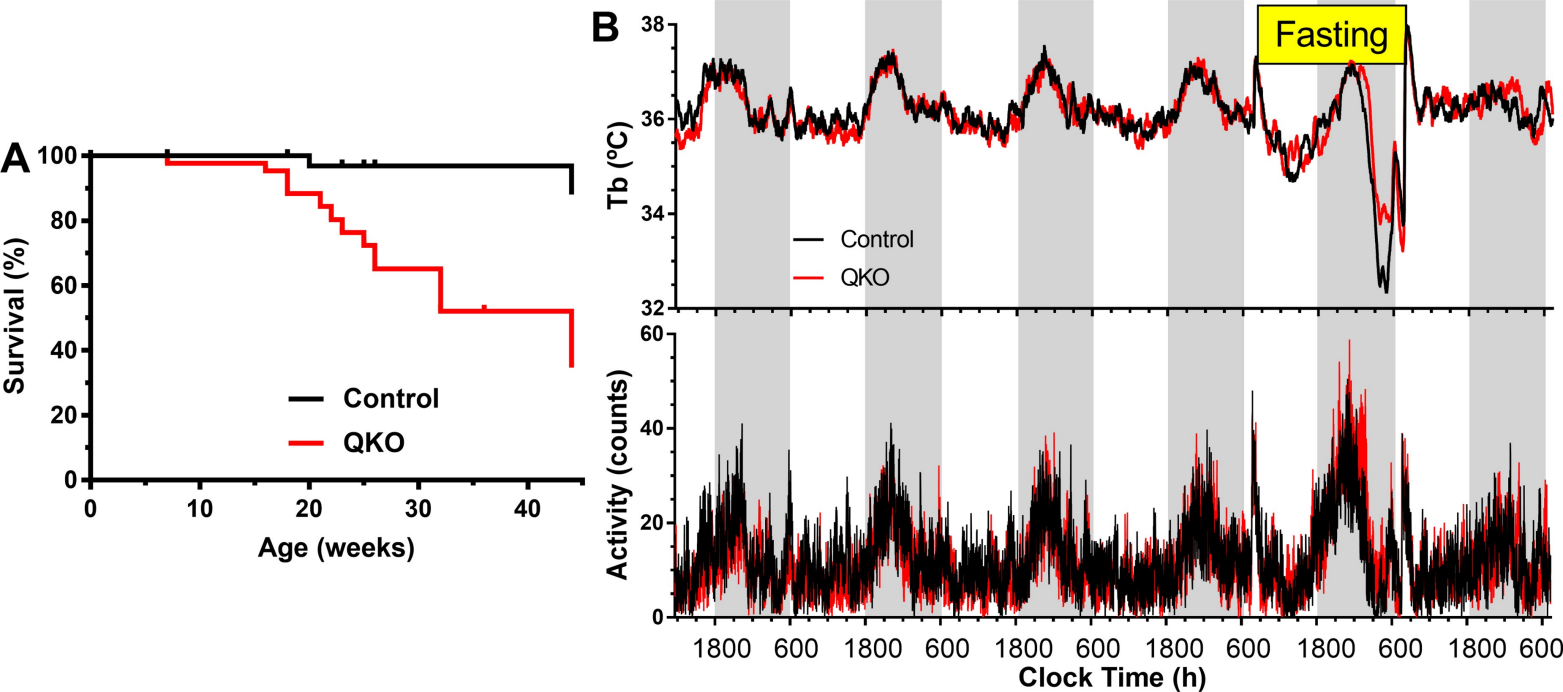
Decreased survival of QKO mice with normal Tb and entry into torpor. A. Kaplan–Meier survival of QKO (*N* = 43) and control (*N* = 45) mice, males and females. QKO survival is less than control, *P* = 0.0005 by Mantel–Cox test. B. QKO and control male mice show similar diurnal rhythm, and both groups enter torpor with a 24-hour fast. Data are means; SEMs omitted for visual clarity; *N* = 11–13/group. The numerical data are in Supporting information. QKO, quad knockout; Tb, core body temperature.

QKO mice (*Adora1*^*−/−*^*;Adora2a*^*−/−*^*;Adora2b*^*−/−*^*;Adora3*^*−/−*^) were produced by crossing the previously characterized individual AR knockout mice [18,24–26] as detailed in Mice in Materials and methods. The *Adora1*^*−*^ allele was slightly underrepresented in the progeny [27,28], without pairwise interaction with genotype at the other three loci. QKO mice at 2 months of age appeared to breed normally, with litter sizes similar to controls (control, 9.8 ± 0.6 per litter, *n* = 11 litters versus QKO 9.6 ± 0.9 per litter, *n* = 5 litters; *P* = 0.85, unpaired *t* test). Breeding of 5-month-old QKO mice produced smaller litters (QKO litters of 1, 6, 3, and 7 pups versus control litters of 8, 9, 10, and 9 pups). In addition, the QKO mice showed decreased survival starting at about 15 weeks of age (Fig 1A). Two independent lines of *Adora1*^*−/−*^ mice also show decreased survival (https://www.jax.org/strain/014161 and George Yuning Huang, National Institute of Diabetes and Digestive and Kidney Diseases, personal communication); to our knowledge, decreased survival has not been reported for *Adora2a*^*−/−*^, *Adora2b*^*−/−*^, or *Adora3*^*−/−*^ mice. The QKO mice exhibited some eye inflammation and dermatitis, like *Adora2a*^*−/−*^ mice (Dorian McGavern, National Institute of Neurological Disorders and Stroke, personal communication).

Growth, metabolism, and blood characteristics were measured in cohorts of male and female mice (summarized in Table 1). At 8–16 weeks of age, chow-fed mice had a slightly lower body weight due to reduced lean, but not fat, mass (S1 Fig and S2 Fig). These mice also showed improved glucose tolerance and lower serum free fatty acid, cholesterol, and insulin-like growth factor-1 (IGF-1) levels (S1 Fig and S2 Fig). Corticosterone levels were similar to controls with intact diurnal rhythm (control versus QKO: 8 AM 26.0 ± 5.2 versus 19.5 ± 4.3 ng/mL and 6 PM 149.6 ± 18.1 versus 103.9 ± 14.3 ng/mL; *n* = 10/group). Blood monocyte and polymorphonuclear leukocyte counts were reduced (S1 Table). Serum chemistries revealed increased alkaline phosphatase and confirmed the low cholesterol levels (S2 Table). On a high-fat diet, QKO mice were remarkably like control mice. An independent cohort of chow-fed older male mice was slightly heavier (body weight, white adipose tissue [WAT], brown adipose tissue [BAT], and liver, which likely accounts for the higher leptin and insulin; S3 Table). In these mice, there was no difference in β-hydroxybutyrate or thyroxine (T4), while triiodothyronine (T3) was increased and corticosterone was reduced. Overall, QKO and control mice showed some phenotypic differences, which were generally neither striking nor present in all cohorts.

**Table 1:** Anatomic and metabolic characteristics of QKO mice.

Characteristics	Female chow,	Male chow,	Male HFD,	Male chow,
	group housed	singly housed	singly housed	singly housed
	*S1 Fig*	*S2 Fig*	*S2 Fig*	*S3 Table*
Age:	*8–16 weeks*	*8–16 weeks*	*8–16 weeks*	*46 weeks*
**Body weight**	⇩	⇩	no Δ	⇧
**Fat mass**	no Δ	no Δ	no Δ	
**Lean mass**	⇩	⇩	no Δ	
**Food intake**		no Δ	no Δ	
**Energy expenditure**		no Δ	no Δ	
Age:	*17–19 weeks*	*17–19 weeks*	*17–19 weeks*	
**GTT AUC**	⇩	⇩	no Δ	
**GTT insulin**	no Δ	no Δ	no Δ	
**ITT AUC**	no Δ	no Δ	no Δ	
**Glucose (non-fasting)**	⇩	no Δ	no Δ	⇩
**Insulin (non-fasting)**	no Δ	no Δ	no Δ	⇧
**Free fatty acids**	⇩	⇩	no Δ	no Δ
**Triglycerides**	no Δ	no Δ	no Δ	no Δ
**Cholesterol**	⇩	⇩	no Δ	⇩
**Leptin**	no Δ	no Δ	no Δ	⇧
**Adiponectin**	no Δ	no Δ	no Δ	no Δ
**Insulin-like growth factor-1**	⇩	⇩	no Δ	
**β-Hydroxybutyrate**				no Δ
**Corticosterone**				no Δ
**T3**			no Δ	⇧
**T4**			no Δ	no Δ
Age:	*37 weeks*	*32 weeks*	*24 weeks*	*46 weeks*
**Body length**	no Δ	no Δ	no Δ	no Δ
**BAT weight**	no Δ	no Δ	no Δ	⇧
**Inguinal WAT weight**	no Δ	no Δ	no Δ	⇧
**Gonadal WAT weight**	no Δ	no Δ	⇩	no Δ
**Liver weight**	no Δ	⇩	no Δ	⇧
**Spleen weight**	no Δ	no Δ	⇧	no Δ

Primary data are in S1 Fig, S2 Fig, and S3 Table. Arrows indicate significant changes (without multiplicity correction) in the indicated direction of the QKO mice; no Δ indicates no significant change. Abbreviations: AUC, area under the curve; BAT, interscapular brown adipose tissue; GTT, glucose tolerance test; HFD, high-fat diet; ITT, insulin tolerance test; QKO, quad knockout; T3, triiodothyronine; T4, thyroxine; WAT, white adipose tissue.

Kidney function (as assessed by serum creatinine and urea nitrogen, S2 Table) were similar in control and QKO mice. Water intake was similar in control (3.71 ± 0.11 g/day, *n* = 16) and QKO (4.00 ± 0.28 g/day, *n* = 17; *P* = 0.36) mice. Necropsies of male, 32-week-old QKO and control mice (three of each) did not reveal any gross differences in the heart, lungs, liver, kidneys, spleen, gastrointestinal tract, testes, epididymis, seminal vesicles, urinary bladder, preputial gland, or brain. Similarly, no histological abnormalities were apparent in kidney (with tubular lipidosis in both control and QKO mice), heart, lung, spleen, WAT, or BAT.

### Normal body temperature and entry into torpor

Control and QKO male mice tolerated surgical implantation of intraperitoneal (i.p.) sensors for core body temperature (Tb) similarly (100% survival in 13 QKO and 12 controls). The light phase Tb, dark phase Tb, and Tb span, a measure of Tb variation, were the same in QKO and control mice (Table 2). Tb and activity had a similar diurnal rhythm in QKO and control mice, and all mice entered torpor when challenged with a 24-hour fast (Fig 1B). QKO and control mice increased Tb and activity similarly in response to handling for food removal (Fig 1B) or the stress of being placed in a cage previously occupied by an unfamiliar male. mRNA levels in WAT and BAT showed variable increases in Ucp1, but not Cidea or Cox8b (S4 Table). These data indicate that ARs are not required for baseline control of Tb, induction of torpor, or bidirectional Tb regulation.

**Table 2:** Tb and physical activity in QKO and control mice.

Characteristics	Control	QKO	*P*
*N*	11	13	
Body weight (g)	24.36 ± 0.40	23.55 ± 0.37	0.16
Tb, dark phase (°C)	36.41 ± 0.11	36.35 ± 0.06	0.60
Tb, light phase (°C)	35.98 ± 0.11	35.93 ± 0.10	0.72
Tb span (°C)	2.46 ± 0.10	2.38 ± 0.17	0.71
Activity, dark phase (beam breaks)	17.83 ± 0.67	16.15 ± 0.51	0.06
Activity, light phase (beam breaks)	12.00 ± 0.73	10.57 ± 0.51	0.13

Data were obtained using G2 E-mitters and are mean ± SEM from a 72-hour time interval. Male mice were 12 weeks old. Tb span is the difference between the 5th and 95th percentiles and is a measure of Tb variation. *P* is from unpaired *t* tests, without correction for multiple tests.

Abbreviations: Tb, core body temperature; QKO, quad knockout.

### Blood pressure and heart rate

We investigated heart rate and blood pressure by telemetry in unanesthetized, freely moving female mice. Implantation of radio transmitters requires unilateral carotid artery occlusion; survival from this surgery was reduced in the QKO mice (5/11 [45%] QKO versus 34/42 [81%] non-QKO mice survived; *P* = 0.017 by χ^2^). The telemetered QKO mice had a higher light phase heart rate and lower dark phase blood pressure, but the small sample size limits the conclusions (Table 3). The usual diurnal effects (dark phase increases in systolic, diastolic, and mean arterial blood pressure (MAP), and in heart rate, Tb, and physical activity) were similar in QKO and control mice. Thus, ARs are not required for diurnal rhythmicity of blood pressure and heart rate.

**Table 3:** Baseline cardiovascular parameters in QKO and control mice.

Parameters	Control dark	QKO dark	*P*	Control light	QKO light	*P*
*N*	5	5				
Heart rate (bpm)	563 ± 17	587 ± 18	0.35	505 ± 11	558 ± 17	**0.031**
Systolic (mmHg)	128 ± 4	117 ± 3	**0.047**	111 ± 3	108 ± 2	0.46
Diastolic (mmHg)	100 ± 3	91 ± 4	0.11	84 ± 2	82 ± 3	0.65
MAP (mmHg)	114 ± 3	105 ± 3	0.057	98 ± 2	96 ± 1	0.47
Tb (°C)	37.06 ± 0.07	36.68 ± 0.14	**0.041**	35.82 ± 0.11	36.01 ± 0.08	0.20
Activity (au)	0.234 ± 0.061	0.130 ± 0.023	0.15	0.072 ± 0.018	0.061 ± 0.003	0.59

Data are mean ± SEM from a 48-hour time interval of continuous recording during a quiet period in the animal facility, measured using HD-X11 transmitters in 24-week-old female mice. Analysis of the individual 12-hour epochs produced the same conclusions. *P* values are from unpaired *t* tests, without correction for multiple tests.

Abbreviations: au, arbitrary unit; bpm, beats per minute; MAP, mean arterial blood pressure; Tb, core body temperature.

### Loss of adenosine-induced hypothermia, bradycardia, and hypotension in QKO mice

**Fig 2:**
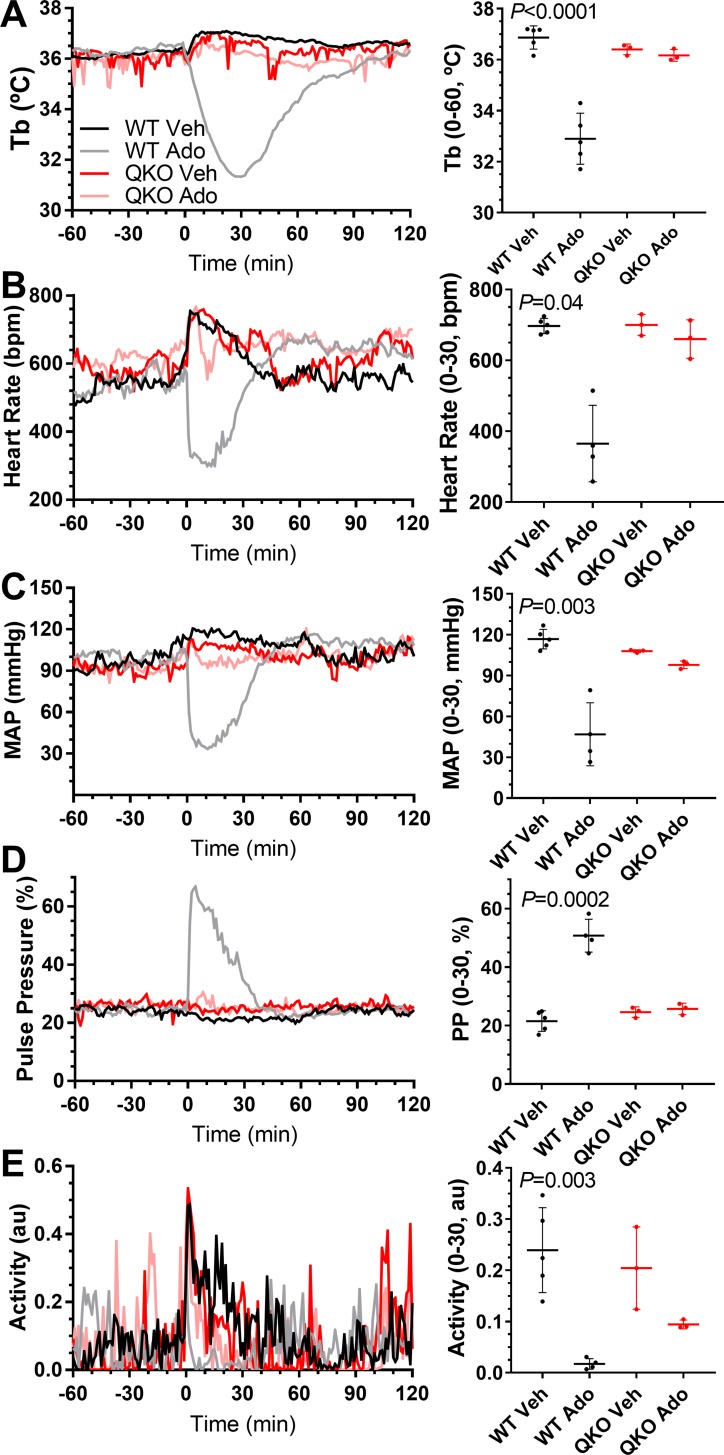
Loss of cardiovascular effects of adenosine in QKO mice. Female QKO and control mice were treated with adenosine (Ado, 100 mg/kg, i.p.) or vehicle (Veh). Adjusted *P* values (from two-way ANOVA with Sidak multiple comparisons test; *N* = 3–5/group) are for effect of vehicle versus adenosine in control mice; adenosine had no significant effect in QKO mice (S6 Table). Activity data were analyzed after log transformation. The numerical data are in Supporting information. Ado, adenosine; au, arbitrary unit; bpm, beats per minute; i.p., intraperitoneal; MAP, mean arterial blood pressure; QKO, quad knockout; Tb, core body temperature; Veh, vehicle; WT, wild-type.

Adenosine causes hypothermia in wild-type mice, via each of the four ARs. The 100 mg/kg dose of adenosine equals about 1/10th of the molar ATP content of the body, so instantaneous conversion of the adenosine (1 adenosine + 2 ATP → 3 ADP) would change a body’s ATP/ADP ratio from 10 to 2 and could signal an energy-depleted state, inducing hypothermia, independent of ARs. When male QKO mice were treated with adenosine, no hypothermia occurred. Similarly, treatment of female QKO mice with adenosine had no clear effect on Tb, heart rate, blood pressure, and activity, while in control mice, adenosine reduced Tb, heart rate, MAP, and activity and increased pulse pressure (Fig 2A–2E). These results demonstrate that adenosine actions at A_1_AR, A_2A_AR, A_2B_AR, and/or A_3_AR mediate the full effects of adenosine-induced hypothermia, bradycardia, and hypotension. This result argues against the hypothesis that pharmacologic dosing of adenosine produces an AR-independent energy-depletion signal.

### Response to lipopolysaccharide

**Fig 3:**
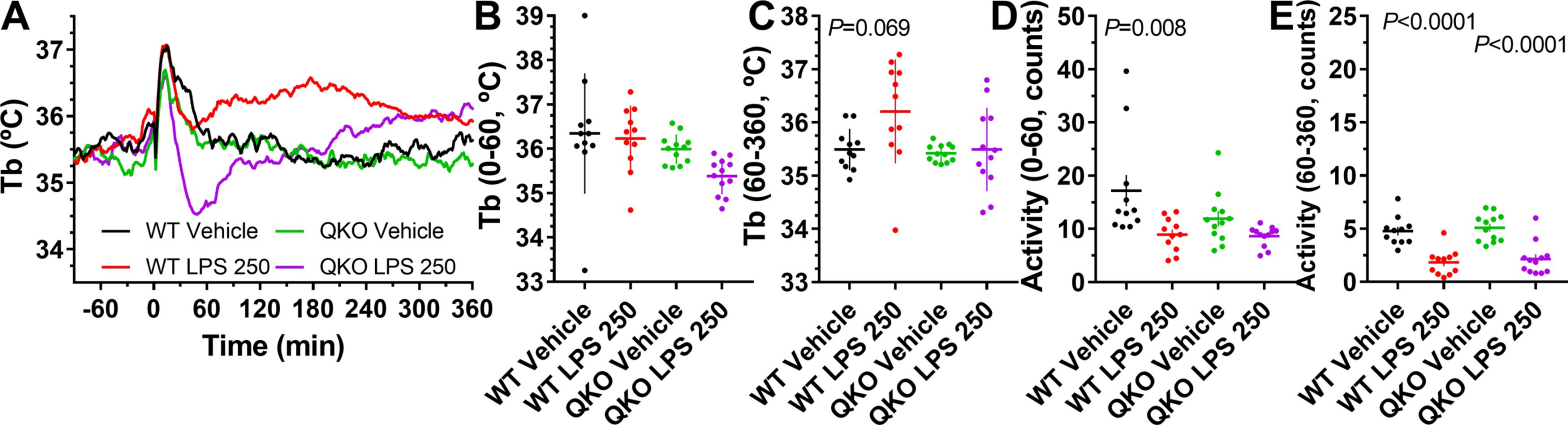
Altered response to LPS in QKO mice. Male QKO and control mice were treated with LPS (250 μg/kg, i.p.) or vehicle. Adjusted *P* values (from two-way ANOVA with Tukey multiple comparisons test; *N* = 11–12/group) are for effect of vehicle versus LPS within genotype (S6 Table). The numerical data are in Supporting information. i.p., intraperitoneal; LPS, lipopolysaccharide; QKO, quad knockout; Tb, core body temperature; WT, wild-type.

Lipopolysaccharide (LPS) stimulates the innate immune system, increasing cytokine levels and causing Tb changes in a dose-dependent manner, with lower doses increasing Tb and higher doses causing hypothermia followed by fever [29,30]. *Adora2b*^*−/−*^ mice are reported to have increased basal and LPS-stimulated cytokine levels [31] and, pharmacologically, A_2A_AR [32] and A_3_AR [18] agonists inhibited LPS-induced cytokine production. We examined the QKO mice to see if their response to LPS was exaggerated. At a low dose (10 μg/kg, i.p.), LPS increased Tb slightly in control (Tb at 60–360 minutes versus vehicle: +0.52 ± 0.19°C, *P* = 0.018) and, similarly, in QKO (+0.58 ± 0.18°C, *P* = 0.007) mice. At a dose of 50 μg/kg, the QKO mice showed less (*P* = 0.015) increase in Tb in this interval (+0.89 ± 0.19°C, *P* = 0.0009 in control versus +0.31 ± 0.12°C, *P* = 0.023 in QKO). At a higher LPS dose (250 μg/kg, i.p.) the control mice demonstrated an increase in Tb, while the QKO mice had a mild early reduction in Tb, followed by a Tb increase (Fig 3A–3E). These data are consistent with a subtly augmented Tb response to LPS in the QKO mice.

We next studied the cytokine response to LPS (250 μg/kg, i.p.) at 2 hours after dosing. Of the 31 analytes with detectable levels, 24 increased upon treatment with LPS. Interestingly, there was no significant effect of genotype or genotype × treatment interaction on plasma levels of any of these 31 proteins (S5 Table). Thus, in the QKO mice we observed neither a change in basal cytokine levels, nor an exaggerated cytokine response to LPS, suggesting that the innate immune system was not activated because of the lack of ARs.

### AMP-induced hypothermia occurs by AR-dependent and AR-independent mechanisms

**Fig 4:**
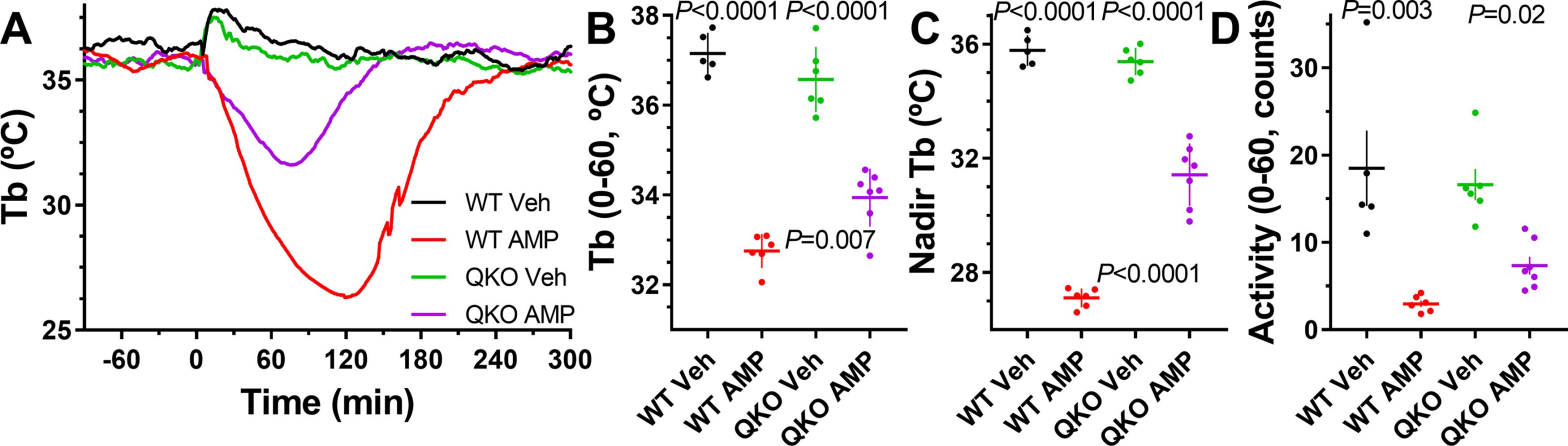
AMP causes less hypothermia in QKO mice compared with controls. Male QKO and control mice were treated with AMP (500 mg/kg, i.p.) or vehicle. Adjusted *P* values (from two-way ANOVA with Tukey multiple comparisons test; *N* = 5–7/group) are (top) for effect of vehicle versus AMP within genotype or (bottom) effect of AMP between genotype (S6 Table). The numerical data are in Supporting information. AMP, adenosine 5′-monophosphate; i.p., intraperitoneal; QKO, quad knockout; Tb, core body temperature; Veh, vehicle; WT, wild-type.

AMP is a proposed natural regulator of torpor, and injection of large doses causes hypothermia [33]. The contribution of ARs to AMP-induced hypothermia is debated [15,34–37]. In the QKO mice, the hypothermia caused by AMP (500 mg/kg, i.p.) was attenuated, but not abolished (Fig 4A–4D). Thus, AMP-induced hypothermia has both AR-dependent and AR-independent components.

### Adenosine kinase inhibition effects are via ARs

**Fig 5:**
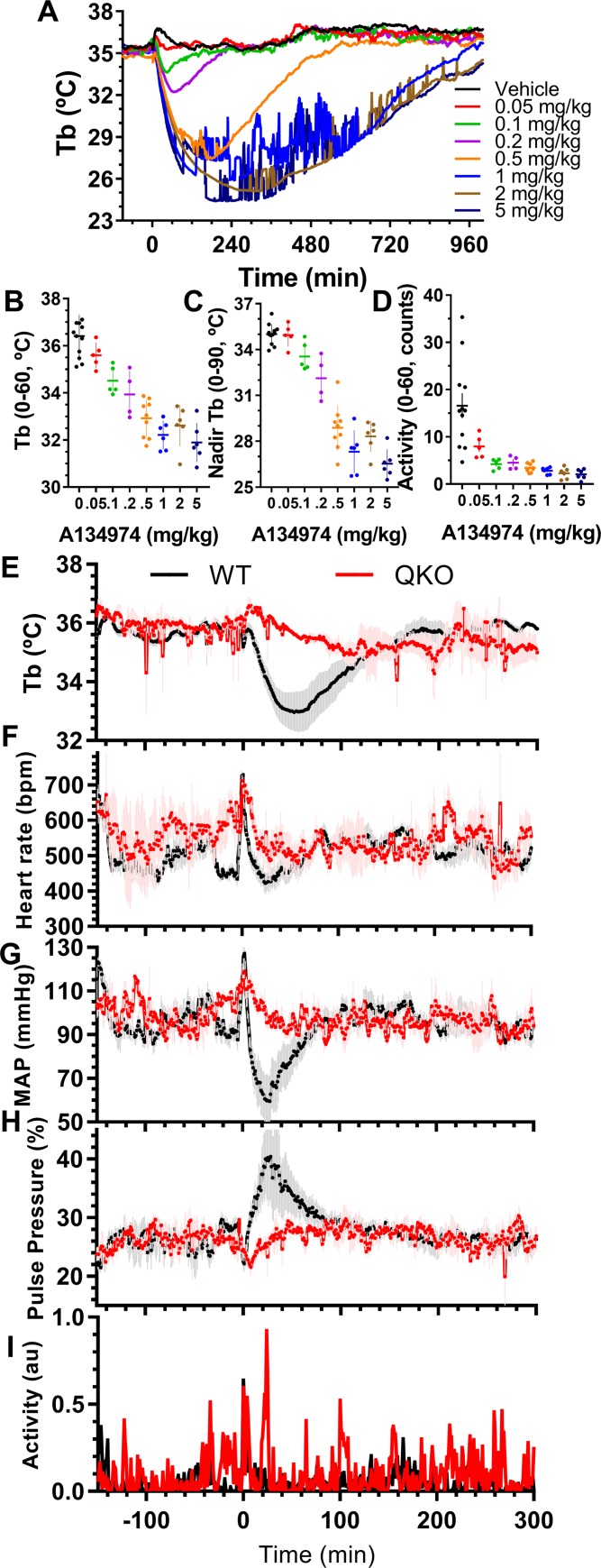
Tb and cardiovascular effects of adenosine kinase inhibition are due to ARs. (A–D) Dose response of A134974 in male QKO and control mice. Smallest doses significantly different (one-way ANOVA with Dunnett multiple comparisons tests; *N* = 4–12/group) from vehicle were 0.1 mg/kg for Tb (0–60), 0.2 mg/kg for nadir Tb, and 0.05 mg/kg for activity (0–60). (E–I) Cardiovascular effects of A134974 in female QKO (5 mg/kg, i.p.) and control (0.5 mg/kg, i.p.) mice (*N* = 3–5/group; statistics in S6 Table). The numerical data are in Supporting information. AR, adenosine receptor; au, arbitrary units; bpm, beats per minute; i.p., intraperitoneal; QKO, quad knockout; Tb, core body temperature; WT, wild-type.

Adenosine kinase phosphorylates adenosine to AMP, keeping intracellular adenosine levels low, thereby drawing extracellular adenosine into cells down its concentration gradient. It has been suggested that inhibition of adenosine kinase causes hypothermia via AR-independent mechanisms [38]. We confirmed that treatment of wild-type mice with the adenosine kinase inhibitor A134974 [39] caused hypothermia in a dose-dependent manner (Fig 5A–5D). In QKO mice, A134974 (5 mg/kg, i.p.) had no effect on heart rate, blood pressure, or Tb, while a 10-fold lower dose caused hypothermia, bradycardia, and hypotension in control mice (Fig 5E–5I). These results suggest that the acute hypothermia and cardiovascular effects of adenosine kinase inhibition are due to increased extracellular adenosine acting on ARs, with no reason to invoke non-AR mechanisms.

### Caffeine effects are lost in QKO mice

**Fig 6:**
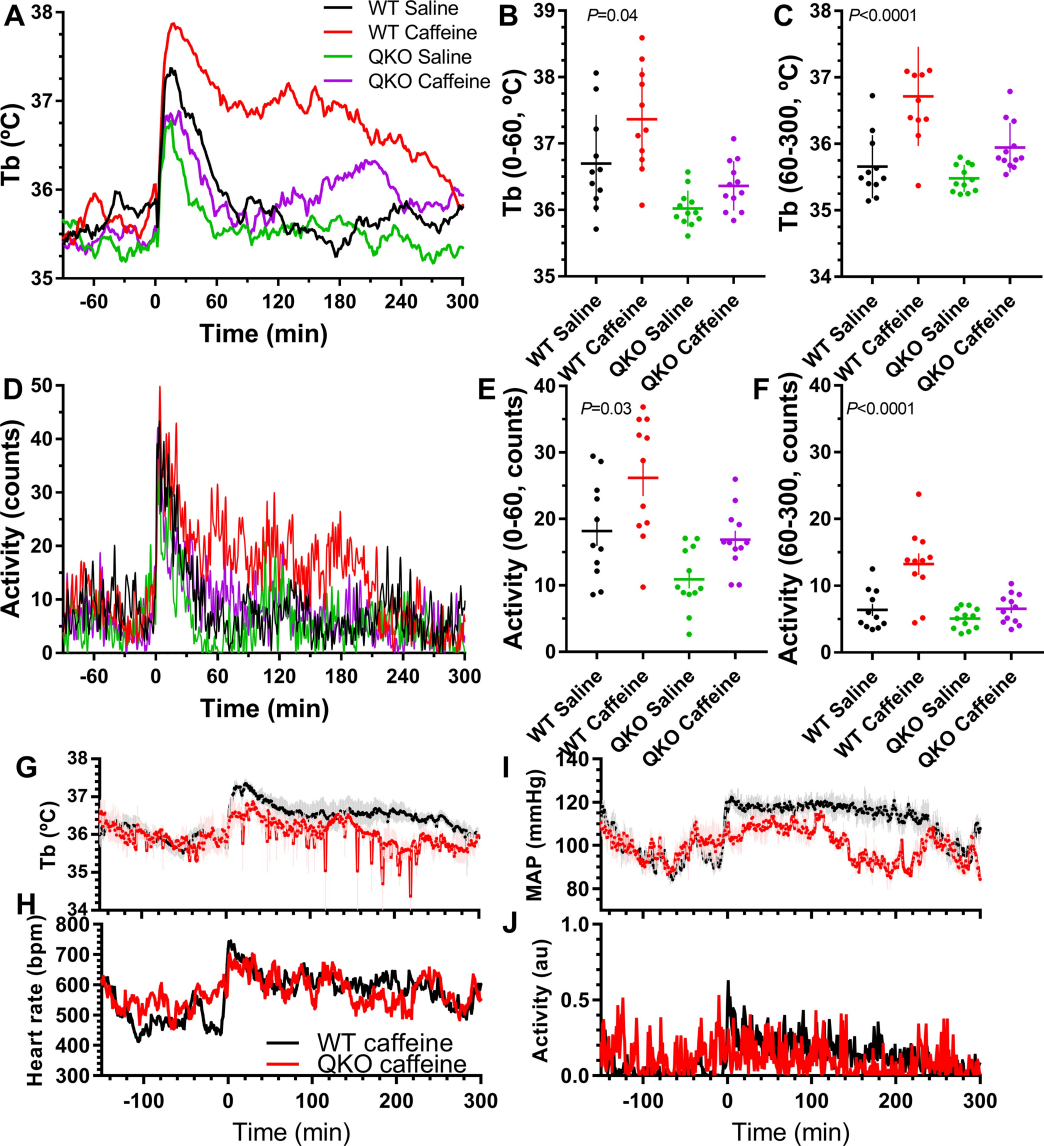
Tb and activity effects of caffeine are lost in QKO mice. (A-F) Male QKO and control mice were treated with caffeine (30 mg/kg, i.p.) or saline. Adjusted *P* values (from two-way ANOVA with Sidak multiple comparisons test; *N* = 11–12/group) are for effect of vehicle versus caffeine in control mice; caffeine effect was not significant in QKO mice. (G-J) Cardiovascular effects of caffeine (30 mg/kg, i.p.) or saline in female QKO mice (*N* = 3–5/group; statistics in S6 Table). The numerical data are in Supporting information. au, arbitrary unit; bpm, beats per minute; i.p., intraperitoneal; MAP, mean arterial blood pressure; QKO, quad knockout; Tb, core body temperature; WT, wild-type.

Caffeine is a brain-penetrant micromolar antagonist of all four human ARs [40] and three of the mouse ARs (not A_3_AR) [41,42]. Caffeine can also act at multiple other sites. In control mice, caffeine (30 mg/kg, i.p.) caused a prolonged increase in Tb, activity, and blood pressure, all of which were attenuated or lost in the QKO mice (Fig 6A–6J). These data suggest that these actions of this dose of caffeine are mediated by ARs.

### Inosine causes hypothermia via mast cell A_3_AR

**Fig 7:**
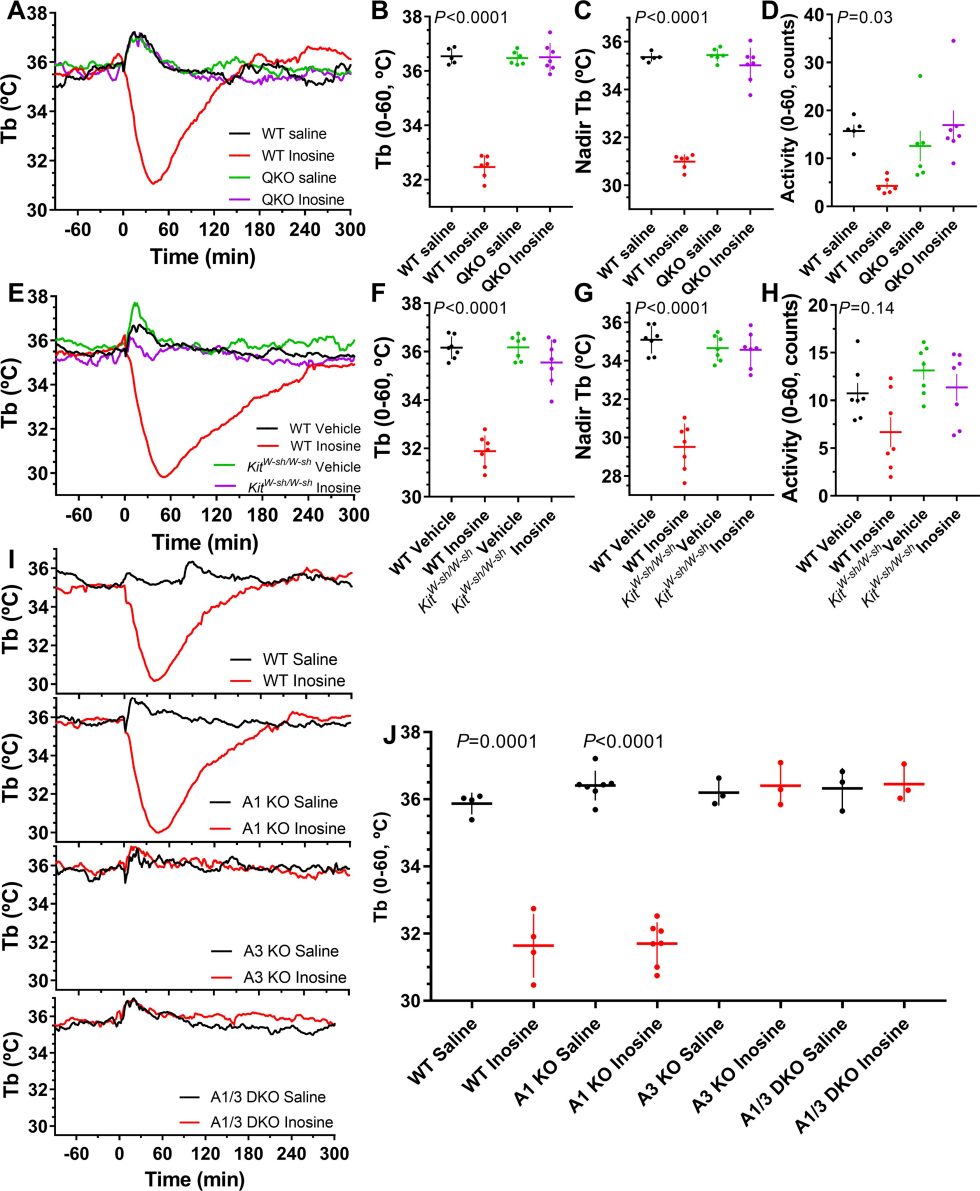
Inosine causes hypothermia via adenosine receptor A_3_AR. (A-D) Effect of inosine (200 mg/kg, i.p.) or saline in male QKO and control mice; *N* = 5–7/group. (E-H) Effect of inosine (200 mg/kg, i.p.) or saline in *Kit*^*W−sh/W−sh*^ and control mice. Adjusted *P* values (from two-way ANOVA with Tukey multiple comparisons test; *N* = 7/group) are for effect of vehicle versus inosine within genotype. (I-J) Effect of inosine (200 mg/kg, i.p.) or saline in control (WT), *Adora1*^*−/−*^ (A1 KO), *Adora3*^*−/−*^ (A3 KO), and *Adora1*^*−/−*^ *Adora3*^*−/−*^ (A1/3 DKO) mice. *P* values (paired *t* test) are for effect of vehicle versus inosine (*N* = 3–7/group; S6 Table). The numerical data are in Supporting information. DKO, double knockout; i.p., intraperitoneal; KO, knockout; QKO, quad knockout; Tb, core body temperature; WT, wild-type.

Inosine is formed by deamination of adenosine and has no known dedicated receptors, but has in vivo actions with multiple proposed mechanisms. Treatment of wild-type mice with inosine (200 mg/kg, i.p.) produced hypothermia and hypoactivity. Both effects were lost in the QKO mice (Fig 7A–7D). The hypothermia was also lost in *Kit*^*W−sh/W−sh*^ mice, which lack mast cells (Fig 7E–7H), and in *Adora3*^*−/−*^ but not *Adora1*^*−/−*^ mice (Fig 7I and 7J). The most parsimonious explanation of the data is that inosine is acting on mast cell A_3_AR, causing mast cell activation, including histamine release and hypothermia [19,43,44]. Inosine is probably not increasing extracellular adenosine by transporter inhibition [45,46], as this would cause hypothermia via other ARs, in addition to A_3_AR (see below).

### Uridine-induced hypothermia requires ARs

**Fig 8:**
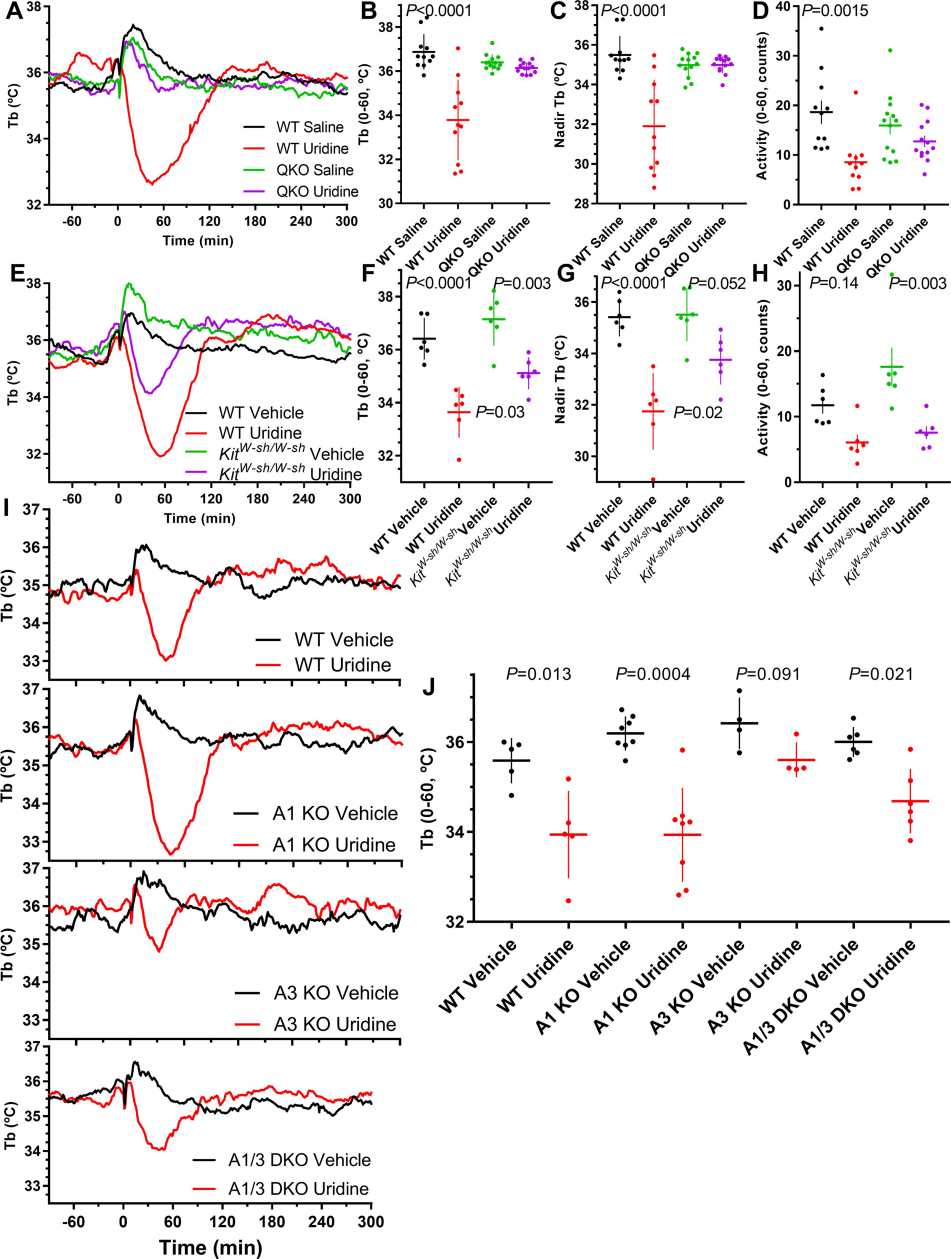
Uridine causes hypothermia via multiple ARs. (A-D) Effect of uridine (1,000 mg/kg, i.p.) or saline in male QKO and control mice; *N* = 11–13/group. (E-H) Effect of uridine (1,000 mg/kg, i.p.) or saline in *Kit*^*W−sh/W−sh*^ and control mice. Adjusted *P* values (from two-way ANOVA with Tukey multiple comparisons test; *N* = 6/group) are for effect of vehicle versus uridine within genotype (S6 Table). (I-J) Effect of uridine (1,000 mg/kg, i.p.) or saline in control (WT), *Adora1*^*−/−*^ (A1 KO), *Adora3*^*−/−*^ (A3 KO), and *Adora1*^*−/−*^*;Adora3*^*−/−*^ (A1/3 DKO) mice. *P* values (unpaired *t* test; *N* = 4–8/group) are for effect of vehicle versus uridine. The numerical data are in Supporting information. AR, adenosine receptor; DKO, double knockout; i.p., intraperitoneal; KO, knockout; QKO, quad knockout; Tb, core body temperature; WT, wild-type.

A recent study found that plasma uridine levels decreased with feeding and increased with fasting and suggested that uridine was linked to the reduction in Tb during fasting [47]. We replicated the effect of exogenous uridine (1,000 mg/kg, i.p.) to cause hypothermia in control mice. However, the uridine did not cause hypothermia in QKO mice (Fig 8A–8D). The uridine-induced hypothermia was distinct from inosine-induced hypothermia, as it was intact in *Adora1*^*−/−*^ mice and attenuated but not eliminated in *Adora3*^*−/−*^, *Adora1*^*−/−*^;*Adora3*^*−/−*^, and *Kit*^*W−sh/W−sh*^ mice (Fig 8E–8J), suggesting action via A_3_AR and also A_2A_AR, and/or A_2B_AR.

**Fig 9:**
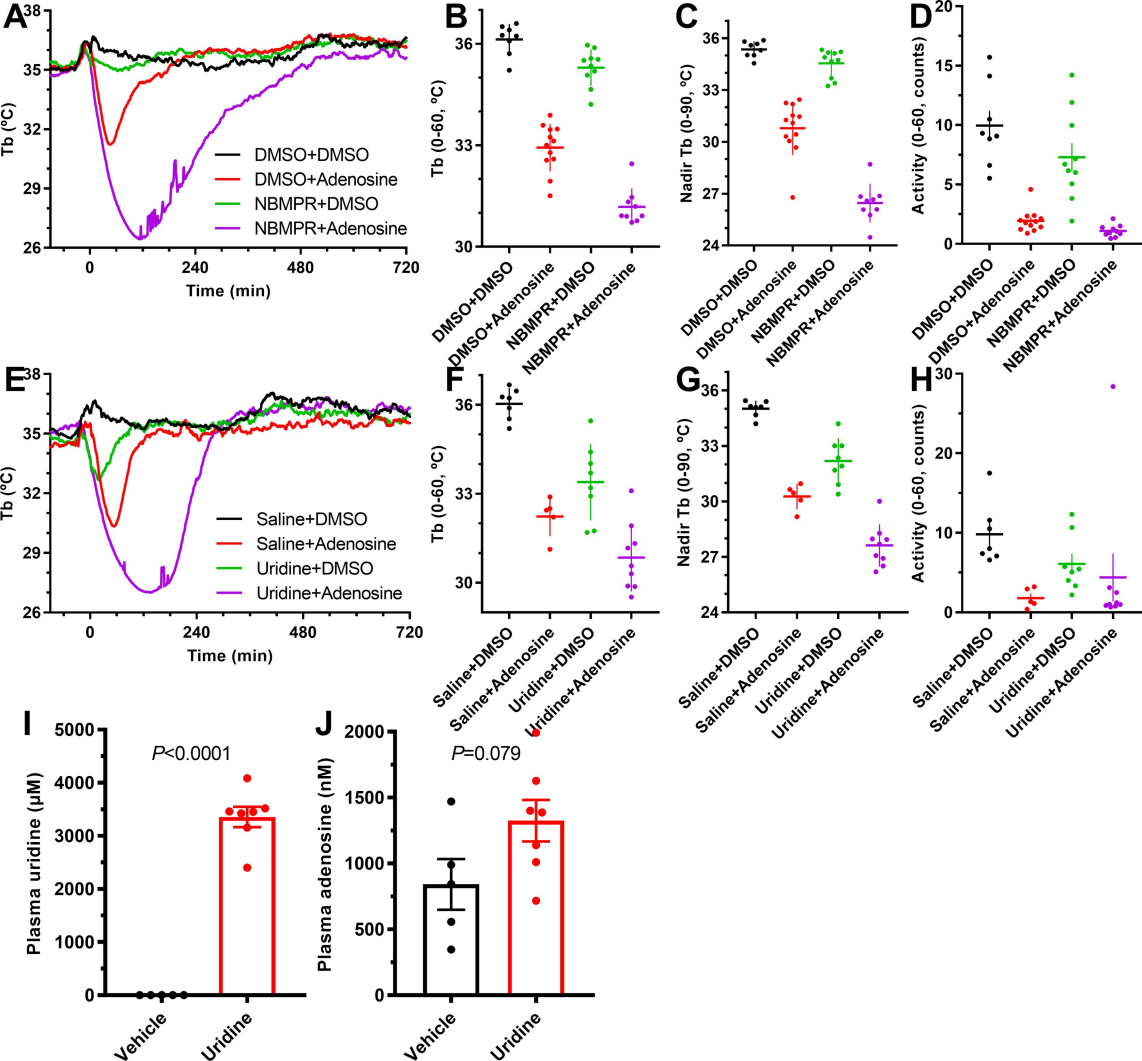
Effect of treatment with ENT1 inhibitor or uridine on hypothermia. (A-D) Effect of the ENT1 inhibitor, NBMPR (1 mg/kg, i.p.), or vehicle on adenosine (100 mg/kg, i.p.) hypothermia in C57BL/6J mice; *N* = 9–12/group. (E-H) Effect of uridine (1,000 mg/kg, i.p.) or saline on adenosine (100 mg/kg, i.p.) hypothermia in C57BL/6J mice. Adjusted *P* values (*N* = 5–9/group) are in S6 Table. (I-J) Plasma uridine and adenosine levels 30 minutes after uridine (1,000 mg/kg, i.p.) or saline in C57BL/6J mice. *P* values (unpaired *t* test; *N* = 5–7/group) are for effect of uridine versus vehicle. The numerical data are in Supporting information. ENT1, equilibrative nucleoside transporter 1; i.p., intraperitoneal; NBMPR, 6-S-[(4-Nitrophenyl)methyl]-6-thioinosine; Tb, core body temperature.

How does uridine cause hypothermia via the ARs? Uridine and adenosine are both substrates for the equilibrative nucleoside transporter 1 (ENT1), competing with each other for transport [45,46]. An ENT1 inhibitor (6-S-[(4-Nitrophenyl)methyl]-6-thioinosine [NBMPR], 1 mg/kg, i.p.) had slight effects on Tb by itself, but greatly amplified the hypothermia caused by adenosine (100 mg/kg, i.p.) (Fig 9A–9D). We identified a uridine dose (1,000 mg/kg, i.p.) with a modest effect on Tb and tested its effect on adenosine-induced hypothermia, finding an increased effect (Fig 9E–9H). We next measured plasma adenosine in uridine-treated (2,000 mg/kg, i.p.) wild-type mice at 30 minutes after dosing, when uridine levels peak [48]. Uridine levels were low (≤2 μM) in vehicle-treated controls and 3,360 ± 190 μM in uridine-treated mice (Fig 9I); uridine treatment tended to increase plasma adenosine levels (0.842 ± 0.086 μM in vehicle-treated versus 1.33 ± 0.06 μM in uridine-treated mice; *P* = 0.08) (Fig 9J). Taken together, these results suggest that uridine inhibits adenosine transporters such as ENT1, thereby increasing extracellular adenosine levels and causing hypothermia via mast cell A_3_AR and other ARs.

## Discussion

We generated QKO mice to examine adenosine physiology and test whether exogenous adenosine-induced hypothermia occurs independent of ARs, such as by energy depletion. For example, brain glucoprivation produces hypothermia [49] and glucose and fatty acids levels are sensed in the hypothalamus [50,51]. Specifically, AMPK is a cellular energy sensor and we hypothesized that uptake and phosphorylation of adenosine might trigger hypothermia such as via AMPK. While we cannot formally rule out complex multihit mechanisms requiring both ARs and non-AR mediators, the most parsimonious interpretation of the lack of adenosine-induced hypothermia in the QKO mice is that adenosine causes hypothermia via ARs and not by cellular energy depletion.

### QKO and adenosine physiology

We were surprised by the baseline phenotype of mice lacking all ARs. We had hypothesized that QKO mice might have a basally activated immune system and not survive or be healthy. Young QKO mice turned out to be viable and breed readily. We did observe reduced blood monocyte and neutrophil levels, but we do not know the mechanism. Like *Adora1*^*−/−*^ mice (https://www.jax.org/strain/014161), they showed decreased survival starting at about 4 months of age, with no identified cause of death. While the QKO mice showed no mortality from the surgery for i.p. implantation of Tb telemeters, they did exhibit increased mortality from surgery for cardiovascular telemeter implantation, which requires ligation of one carotid artery. A crucial difference is that carotid ligation is an ischemic event, increasing brain adenosine, which is neuroprotective via multiple ARs [52]. These protective effects cannot occur in the QKO mice, confirming adenosine’s beneficial effects in certain “danger” situations.

While LPS treatment showed a subtly modified effect on Tb, the QKO mice had cytokine levels (basal and LPS-stimulated) that were the same as those in control mice. This differs from the reported elevated basal and LPS-stimulated TNF-α and IL-6 levels in *Adora2b*^*−/−*^ mice [31]. Possible explanations for this difference include LPS dose, genetic background, and effect of the other AR genotypes. However, most literature on the role of ARs in modulating the LPS response examines the effect of exogenous drugs [18,32,44,53], not of AR loss. Basal cytokine levels were reported unchanged with loss of A_2A_AR [32] or A_3_AR [18]. This evidence again suggests that adenosine is more important for “emergency physiology” in danger/damage situations, and not under basal conditions.

Adenosine physiology can be difficult to study. Adenosine, the natural ligand, is locally cleared in a few seconds [54], with a plasma half-life of about 1 minute in humans [55]. The rapid clearance means that experimentally, massive adenosine doses are used, with a complicated relationship to endogenous physiology. One solution is use of synthetic ligands, which increase the effect duration and likely provide supraphysiologic activation. It is not known if AR physiology from transient adenosine binding differs from the prolonged activation from agonist ligands. Some synthetic ligands are selective for one AR, but ligand selectivity can vary by >1,000-fold between species [41]. The use of knockout mice is a powerful tool for ensuring ligand selectivity, with loss of drug effect in the null being strong evidence for an “on-target” action, due to the identified AR. However, QKO mice develop from conception without any ARs, which may elicit compensatory mechanisms, underestimating the role of the missing AR. Therefore, a more prominent phenotype of the QKOs might appear in different experimental stress models. Finally, ARs can heterodimerize with other G protein-coupled receptors and use multiple signaling routes [56], so it is possible that probing adenosine effect may require understanding of dimerization partners and differential effects on signaling pathways.

### Role of adenosine in hypothermia induced by various nucleosides and their modulators

The clear result that adenosine-induced hypothermia is absent in QKO mice makes these mice extremely valuable for investigating the possible role of adenosine in physiology thought to be caused by other agents. For example, the AR contribution to AMP-induced hypothermia is controversial [15,34–37]. The attenuation of AMP-induced hypothermia in the QKO mice demonstrates that both AR-independent and AR-dependent mechanisms contribute. Additionally, the QKO mice enable study of the non-AR mechanism.

In contrast, the QKO mice demonstrate that the acute hypothermia caused by an adenosine kinase inhibitor is completely attributable to ARs. Some of the hypothermia is due to the increased extracellular adenosine activating A_1_AR [38]. While possible explanations for the remaining A134974-induced hypothermia have been proposed, the QKO results dictate that the non–A_1_AR-mediated hypothermia must occur via A_2A_AR, A_2B_AR, and/or A_3_AR.

Inosine does not have identified dedicated receptors. Mechanisms that might explain inosine’s neuroprotective and immunomodulatory actions include all four ARs and non-AR routes (reviewed in [57]). Inosine can activate A_3_AR on mouse mast cells [43,58]. Inosine has also been reported to be an agonist at A_1_AR [59], A_2A_AR [60,61], and A_2B_AR [62]. Our QKO and follow-up experiments demonstrate that inosine-induced hypothermia is due to A_3_AR agonism.

Caffeine is an AR antagonist [40,41]. At higher concentrations, it inhibits phosphodiesterases [63], γ-aminobutyric acid A (GABA_A_) receptors [63], and glycine receptors [64] and activates ryanodine receptors [65]. We examined a 30 mg/kg dose, which approximates the human plasma exposure of 2–3 cups of coffee [66]. Our data confirm that the effects of caffeine (30 mg/kg) to increase Tb and activity are mediated by ARs, likely A_2A_AR [42,67,68].

The protective effects of guanosine in the central nervous system (CNS) have been studied, revealing apparent adenosine-dependent and -independent effects, but a guanosine receptor has not been identified [69–71]. While more mechanistic studies of guanosine’s effects are warranted, we were unable to explore this with QKO mice, because a high, solubility-limited dose of guanosine (60 mg/kg, i.p.) [70] failed to lower Tb in wild-type mice.

### Mechanism of uridine-induced hypothermia

We were surprised by the QKO results demonstrating that uridine-induced hypothermia requires ARs. Uridine (at doses reaching ≥10 mM in plasma) causes hypothermia in mice and rats [48]. A recent study found that physiological plasma uridine levels decreased with feeding and increased with fasting (albeit in a much lower range, 4–10 μM) and suggested that uridine was linked to the reduction in Tb that occurs during fasting [47]. The result that uridine-induced hypothermia is absent in QKO mice caused us to investigate further. Uridine itself does not bind to ARs [72], is not a substrate for adenosine kinase [73], and does not block adenosine deaminase [74]. However, uridine is a substrate for transport by ENT1 and a competitive inhibitor of adenosine transport by ENT1 [45, 46], and we showed that uridine mimicked the effects of the selective ENT1 inhibitor, NBMPR. Thus, we propose that the pharmacologic effects of uridine are due at least in part to inhibition of ENT1, preventing adenosine uptake into cells. The increased extracellular adenosine activates multiple ARs (mast cell A_3_AR and other ARs). This may explain the sleep-promoting and anticonvulsant effects of uridine (reviewed in [75]). It seems likely that this AR-dependent mechanism for Tb regulation with large exogenous uridine doses does not explain Tb effects of changes of uridine levels in the physiologic range.

### AR-independent effects of adenosine

The QKO mice described here are a valuable research tool to study AR-independent effects of adenosine. Adenosine is an obligatory product of S-adenosylmethionine (SAM)–dependent transmethylation reactions, which include DNA methylation and histone methylation. Through mass action, adenosine provides negative feedback control for transmethylation—low levels of adenosine drive DNA methylation, whereas high levels of adenosine block DNA methylation [76]. The QKO mice would be an ideal investigative tool to study the epigenetic mechanisms of adenosine in the absence of potentially confounding ARs.

### Limitations

We monitored growth and metabolic parameters, Tb, activity, and cardiovascular end points. Other assays might expose additional differences in the QKO mice. While the mice have a mixed genetic background, both QKO and control mice were bred using a scheme to minimize founder/inbreeding effects. Lastly, mice with germline AR deletion may have undergone adaptive changes during development, masking deficits due to loss of ARs [77,78]. Use of conditional AR alleles with deletion in adulthood would avoid this potential confound.

### Implications

We have demonstrated that the physiology elicited by adenosine, uridine, inosine, caffeine, and (partially) AMP requires ARs. It is notable that the QKO mouse phenotype replicates that of individual AR knockout mice, without evidence for synergy, suggesting that the actions of the four ARs are generally complementary. The phenotype of the QKO mice supports the hypothesis that extracellular adenosine has less of a contribution to baseline physiology and is likely more important for its roles as a signal of stress, damage, and/or danger.

## Materials and methods

### Ethics statement

Studies were approved by the Animal Care and Use Committee of National Institute of Diabetes and Digestive and Kidney Diseases, animal protocol K016-DEOB-17. Mice were anesthetized with isoflurane or ketamine and xylazine. Euthanasia of anesthetized mice was performed by exsanguination via retro-orbital bleeding followed by cervical dislocation.

### Mice

Male C57BL/6J and *Kit*^*W−sh/W−sh*^ (JAX 012861) mice were obtained from the Jackson Laboratory. *Adora1*^*−*/*−*^ [24], *Adora2b*^*−*/*−*^ [26] (JAX 022499), and *Adora3*^*−*/*−*^ [18] mice were on a C57BL/6J genetic background. The *Adora2a*^*−*/*−*^ mice [25] (JAX 010685) were on a mixed background. Mice were genotyped as described (S3 Fig) [15,21]. Mice were singly housed at about 22°C with a 12:12-hour light–dark cycle. Chow (NIH-07, Envigo, Madison, WI) or high-fat diet (D12492, 60% kcal fat, 5.24 metabolizable kcal/g; Research Diets) and water were available ad libitum. Mice were studied ≥7 days after any operation or prior treatment. Reuse of mice tends to reduce physical activity levels, presumably due to acclimation. No specific effort was made to acclimate mice to handling in individual experiments.

To generate QKO mice, mice heterozygous at all four loci (*Adora1*^+/*−*^;*Adora2a*^+/*−*^;*Adora2b*^+/*−*^;*Adora3*^+/*−*^) were crossed. Of 334 progeny (310 successfully genotyped at all four loci), the *Adora2a*, *Adora2b*, and *Adora3* alleles were transmitted in the expected mendelian ratios. The *Adora1*^*−*^ allele was slightly underrepresented (+/+:+/−:−/−, observed 109:146:65 versus expected 80:160:80, χ^2^ *P* = 0.0007), as observed previously [27,28]. There was no pairwise interaction of the *Adora1*^*−*/*−*^ genotype with genotype at the other three loci (the mice with *Adora1*^*−*/*−*^ genotype had the expected 1:2:1 +/+:+/−:−/− ratios for each of the *Adora2a*, *Adora2b*, and *Adora3* loci). The QKO mice studied were the second- or third-generation progeny from the quad heterozygote intercross. Controls were +/+ or +/− at the four AR loci and are also second- or third-generation progeny of quad heterozygote intercross.

### Compounds

Compounds (source; vehicle) were obtained as follows: 5′-AMP (Sigma A1752; saline), adenosine (Sigma; 10% DMSO in saline), uridine (Sigma 1707114; saline), A134974 (Sigma A2846; saline), caffeine (Sigma; saline), dipyridamole (Sigma; 10% DMSO in saline), EHNA (erythro-9-(2-Hydroxy-3-nonyl)adenine hydrochloride, Tocris 1261; 10% DMSO; 10 mg/kg dose chosen to avoid phosphodiesterase inhibition [79]), inosine (Sigma I4125; saline; dose based on [80]), NBMPR (6-S-[(4-Nitrophenyl)methyl]-6-thioinosine, Tocris 2924; warm 10% DMSO in saline), and LPS (Sigma L6511; saline).

### In vivo physiology

Tb and activity were continuously measured by telemetry, using i.p. G2 E-mitters, ER4000 energizer/receivers, and VitalView software (Starr Life Sciences, Oakmont, PA), with data collected each minute [81]. Average Tb over 0–60 minutes and nadir Tb during 0–90 minutes after drug injection were used to quantitate hypothermic effects. Average activity over 0–60 minutes captures the reduced activity that accompanies hypothermia. Total minutes <34°C, a better measure of prolonged hypothermia, did not add useful information. Inhibitors were dosed 20–25 minutes before agonists.

Continuous ambulatory intra-arterial blood pressure, heart rate, physical activity, and Tb were measured with radio transmitters (HD-X11, Data Sciences International, St Paul, MN) implanted under ketamine and xylazine anesthesia in the carotid artery as described [82]. Data were sampled at 1,000 Hz using a PhysioTel RPC-1 receiver and Ponemah v6.30 (Data Sciences International) software, with 1-minute averages used for analysis. The pulse pressure is the difference between the systolic and diastolic blood pressure, expressed as a percentage of the systolic pressure.

The following assays were used: β-hydroxybutyrate (colorimetric, #K632, BioVision), corticosterone (RIA, #07120102, MP Biomedicals), T3 (ELISA, #T3043T-100, Calbiotech) and T4 (ELISA, #T4044T-100, Calbiotech). Cytokines were measured in plasma obtained 2 hours after dosing [83] LPS (250 μg/kg, i.p.) or vehicle using the Mouse Magnetic Luminex Assay Kit (LXSAMSM, R&D Systems), performed by the NHLBI Flow Cytometry Core.

Plasma adenosine and uridine measurement: 30 minutes after dosing with uridine or vehicle, C57BL/6J mice were anesthetized with isoflurane, and blood was drawn retro-orbitally into an equal volume of ice-cold stop solution (5 μM EHNA, 200 μM dipyridamole, 4 mM EDTA in saline) to prevent generation or loss of adenosine [84,85]. Samples were centrifuged (4°C, 13,000 rpm, 3 minutes), 50 μL of plasma was pipetted into 200 μL of methanol containing internal standards (^13^C_9_,^15^N_2_-uridine and ^13^C_5_-adenosine, Cambridge Isotopes), vortexed (5 minutes), centrifuged (4°C, 14,000 rpm, 10 minutes), and the supernatant was dried at room temperature under nitrogen and resuspended in 125 μL H_2_O. Samples were analyzed by UPLC-MS/MS using a Vanquish UPLC (Thermo Scientific) and Altis LC-Triple quadrupole mass spectrometer (Thermo Scientific) with heated electrospray ionization (HESI-II, Thermo Scientific) in positive ion mode (3,500 V). Injection volume was 1 μL (autosampler at 5°C), using a Waters Cortecs C18 + 2.7 μm, 2.1 × 100 mm UPLC column (35°C) at 350 μL/minute with solvent A (0.1% formic acid in H_2_O) and solvent B (0.1% formic acid in methanol). Initially, 99% solvent A for 0.25 minutes, linearly changing to 10% A at 2.15 minutes, remaining at 10% A until 3 minutes, linearly changing to 99% A by 3.5 minutes, and stabilizing for 3 minutes. Transitions quantitated were (m/z): adenosine, 268→119 and 268→136; ^13^C_5_-adenosine 273→119 and 273→136; uridine, 245→96 and 245→113; and ^13^C_9_,^15^N_2_-uridine, 256→101 and 256→119. Adenosine was calibrated (*R*^2^ ≥ 0.999) from 1.0 to 2,500 ng/mL and uridine from 0.25 to 750 μg/mL in the original samples. Literature plasma adenosine levels are 100 to 1,000 nM in human [54] and 145 to 6,100 nM in mouse [38,86,87].

Metabolic phenotyping, including glucose and insulin tolerance tests and hormone and metabolite profiles, were performed as previously described [88].

Complete blood count with differential and serum chemistry tests were performed by the Department of Laboratory Medicine, NIH Clinical Center.

Statistical analyses were all two tailed, performed using Prism, and are detailed in S6 Table.

## Supporting information

S1 Fig Phenotype of chow-fed female QKO and control mice, group housed. QKO mice showed reduced body weight due to decreased lean mass at younger age (8–19 weeks, A-C), reduced fed glucose, improved glucose tolerance without changes in insulin tolerance test and insulin levels (D-J). QKO mice also had lower serum FFA, TG, and cholesterol, as well as IGF-1 levels (K-P). At euthanasia (37 weeks), there was no difference in body weight, body length, and organ weights (Q-T). FFA, free fatty acid; IGF-1, insulin-like growth factor 1; QKO, quad knockout; TG, triglyceride.
(PDF)

S2 Fig Phenotype of male QKO and control mice. Mice were singly housed, fed either on chow or a HFD starting at 8 weeks of age. QKO mice had lower body weight when young (8–16 weeks) due to reduced lean mass on both chow and HFD (A-C). Chow-fed QKO mice had improved glucose tolerance without changes in fed glucose and insulin levels (D-H), reduced levels of FFA, cholesterol, and IGF-1 (P-U). Spleen weight was increased in HFD-fed mice (O, rightmost panel) and trending increase in chow-fed mice (N, rightmost panel). HFD-fed QKO mice showed no clear changes compared with controls (I-M, O first three panels, V-AC). Numerical data are in Supporting information. FFA, free fatty acid; HFD, high-fat diet; QKO, quad knockout.
(PDF)

S3 Fig Genotyping of Adora1, Adora2a, Adora2b, and Adora3 alleles. Protocols are in [15,17] and references therein. The spurious band in *Adora2a* genotyping can be eliminated by using a hot start protocol.
(PDF)

S1 Data Underlying data for Fig 1 in GraphPad Prism v7. (PZFX)

S2 Data Underlying data for Fig 2 in GraphPad Prism v7. (PZFX)

S3 Data Underlying data for Fig 3 in GraphPad Prism v7. (PZFX)

S4 Data Underlying data for Fig 4 in GraphPad Prism v7. (PZFX)

S5 Data Underlying data for Fig 5 in GraphPad Prism v7. (PZFX)

S6 Data Underlying data for Fig 6 in GraphPad Prism v7. (PZFX)

S7 Data Underlying data for Fig 7 in GraphPad Prism v7. (PZFX)

S8 Data Underlying data for Fig 8 in GraphPad Prism v7. (PZFX)

S9 Data Underlying data for Fig 9 in GraphPad Prism v7. (PZFX)

S10 Data Underlying data for S1 Fig in GraphPad Prism v7. (PZFX)

S11 Data Underlying data for S2 Fig in GraphPad Prism v7. (PZFX)

S1 Table Hematology in control and QKO mice. QKO, quad knockout.
(PDF)

S2 Table Serum chemistries in control and QKO mice. QKO, quad knockout.
(PDF)

S3 Table Related to Table 1. Phenotype of older male mice.
(PDF)

S4 Table Adipose tissue mRNA levels. (PDF)

S5 Table Cytokine response to LPS in QKO and control (WT) mice. LPS, lipopolysaccharide; QKO, quad knockout; WT, wild-type.
(PDF)

S6 Table Statistical results in excel. (XLSX)

## Abbreviations

AMP: adenosine 5′-monophosphate
AMPK: adenosine monophosphate–activated protein kinase
AR: adenosine receptor
BAT: brown adipose tissue
bpm: beats per minute
CNS: central nervous system
ENT1: equilibrative nucleoside transporter 1
IGF-1: insulin-like growth factor-1
i.p.: intraperitoneal
LPS: lipopolysaccharide
MAP: mean arterial blood pressure
NBMPR: 6-S-[(4-Nitrophenyl)methyl]-6-thioinosine
QKO: quad knockout
SAM: S-adenosylmethionine
Tb: core body temperature
T3: triiodothyronine
T4: thyroxine
WAT: white adipose tissue
