# Supplementary material for: Physiology and effects of nucleosides in mice lacking all four adenosine receptors
Source: PLoS Biol. 2019 Mar 1;17(3):e3000161. doi: 10.1371/journal.pbio.3000161 (PMC6415873; doi:10.1371/journal.pbio.3000161)
Supplement: S1 Fig — QKO mice showed reduced body weight due to decreased lean mass at younger age (8–19 weeks, A-C), reduced fed glucose, improved glucose tolerance without changes in insulin tolerance test and insulin levels (D-J). QKO mice also had lower serum FFA, TG, and cholesterol, as well as IGF-1 levels (K-P). At euthanasia (37 weeks), there was no difference in body weight, body length, and organ weights (Q-T). FFA, free fatty acid; IGF-1, insulin-like growth factor 1; QKO, quad knockout; TG, triglyceride. (PDF) [file pbio.3000161.s001.pdf]

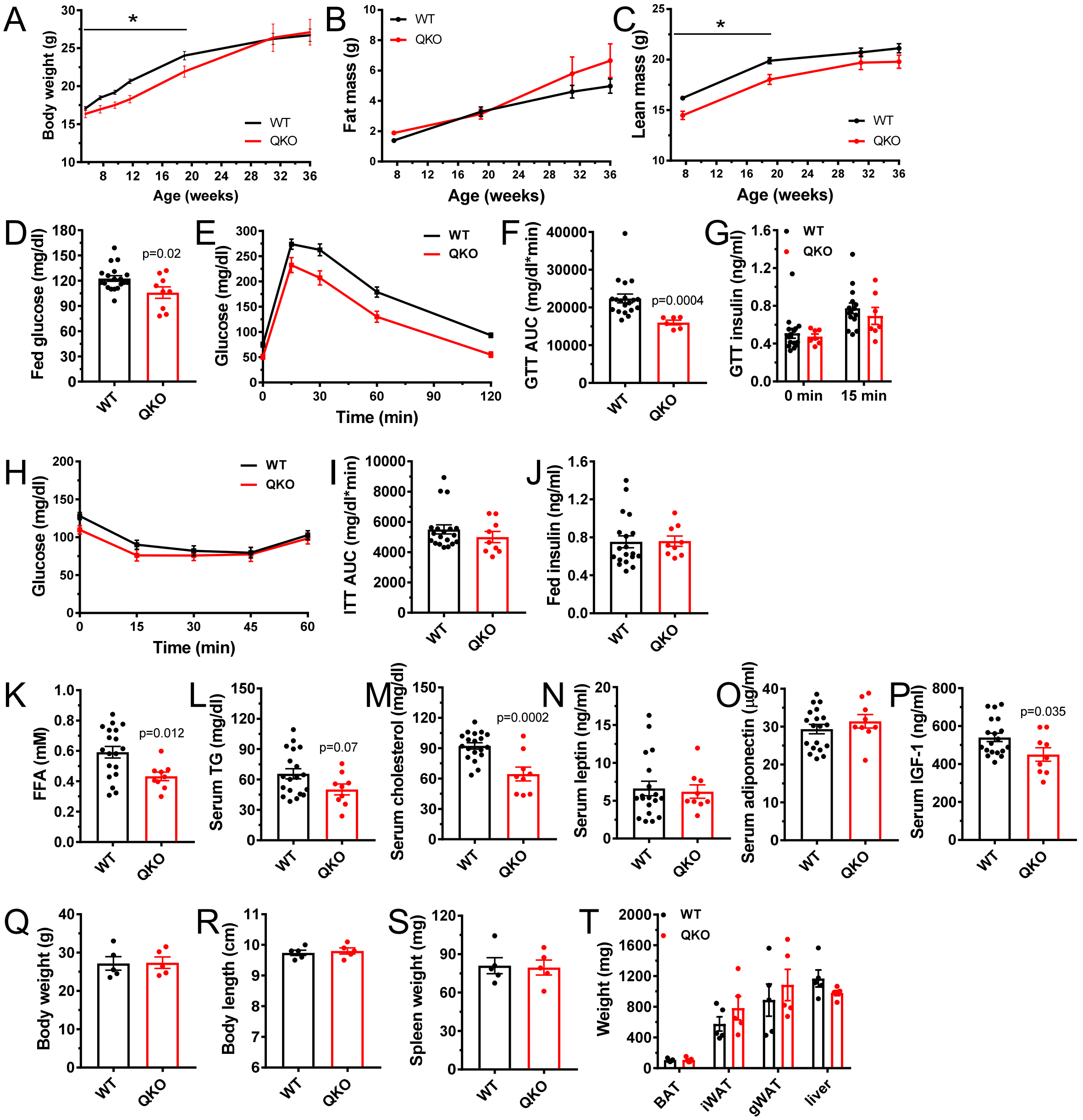

Figure S1: Related to table 1. Phenotype of chow-fed female QKO and control mice, group housed. QKO mice showed reduced body weight due to decreased lean mass at younger age (8-19 weeks, A-C), reduced fed glucose, improved glucose tolerance without changes in insulin tolerance test and insulin levels (D-J). QKO mice also had lower serum free fatty acids (FFA), triglyceride (TG) and cholesterol, as well as insulin-like growth factor1 (IGF-1) levels (K-P). At euthanasia (37 weeks), there was no difference in body weight, body length and organ weights (Q-T). Numerical data are in Supplementary Information.
