# Supplementary material for: Physiology and effects of nucleosides in mice lacking all four adenosine receptors
Source: PLoS Biol. 2019 Mar 1;17(3):e3000161. doi: 10.1371/journal.pbio.3000161 (PMC6415873; doi:10.1371/journal.pbio.3000161)
Supplement: S2 Fig — Mice were singly housed, fed either on chow or a HFD starting at 8 weeks of age. QKO mice had lower body weight when young (8–16 weeks) due to reduced lean mass on both chow and HFD (A-C). Chow-fed QKO mice had improved glucose tolerance without changes in fed glucose and insulin levels (D-H), reduced levels of FFA, cholesterol, and IGF-1 (P-U). Spleen weight was increased in HFD-fed mice (O, rightmost panel) and trending increase in chow-fed mice (N, rightmost panel). HFD-fed QKO mice showed no clear changes compared with controls (I-M, O first three panels, V-AC). Numerical data are in Supporting information. FFA, free fatty acid; HFD, high-fat diet; QKO, quad knockout. (PDF) [file pbio.3000161.s002.pdf]

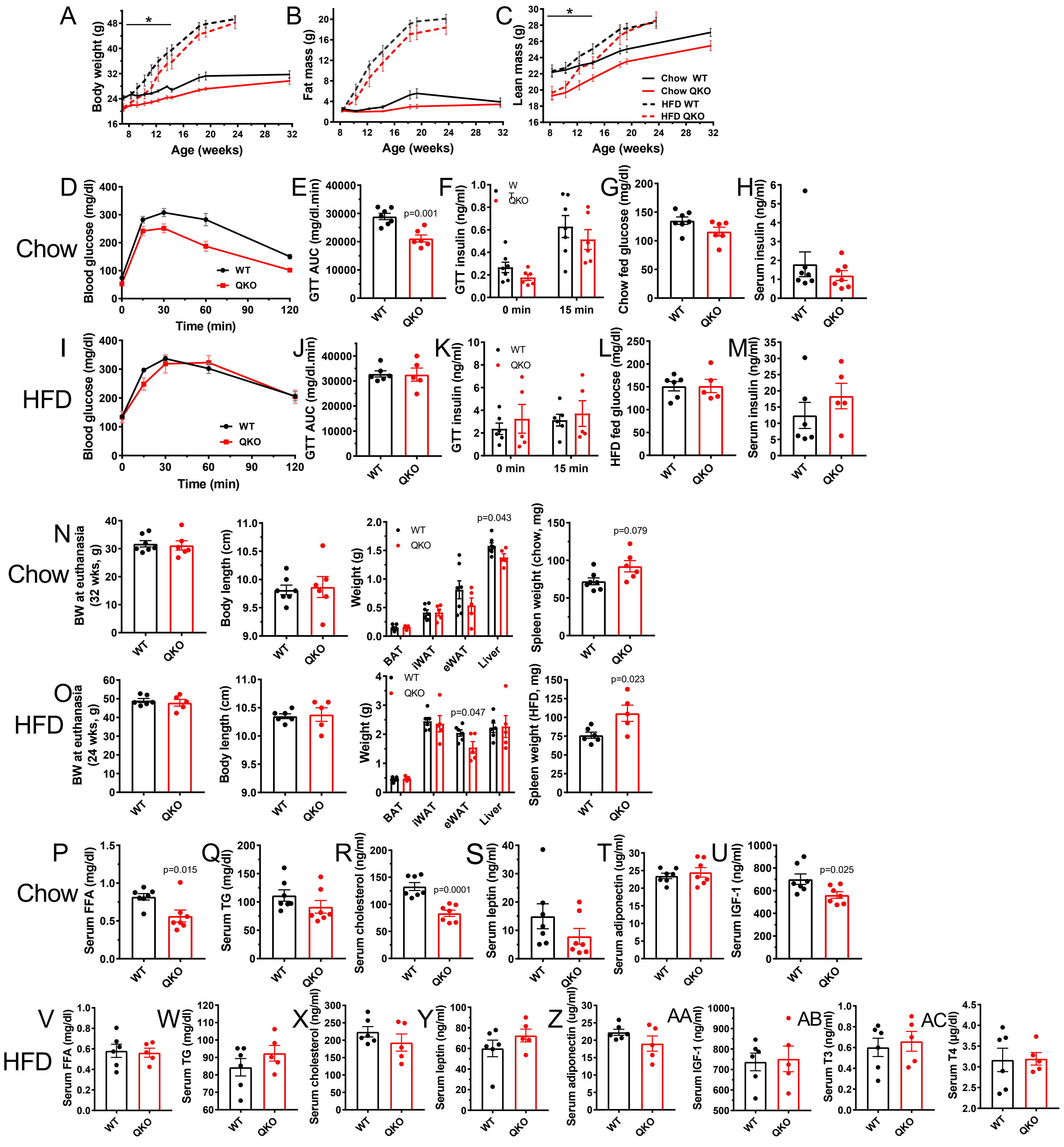

Figure S2. Phenotype of male QKO and control mice. Mice were singly housed, fed either on chow or a high fat diet (HFD) starting at 8 weeks of age. QKO mice had lower body weight when young (8-16 weeks) due to reduced lean mass on both chow and HFD (A-C). Chow-fed QKO mice had improved glucose tolerance without changes in fed glucose and insulin levels (D-H), reduced levels of free fatty acids (FFA), cholesterol and IGF-1 (P-U). Spleen weight was increased in HFD-fed mice (O, right most panel) and trending increase in chow-fed mice (N, right most panel). HFD-fed QKO mice showed no clear changes compared to controls (I-M, O first three panels, V-AC). Numerical data are in Supplementary Information.
