# Supplementary material for: Physiology and effects of nucleosides in mice lacking all four adenosine receptors
Source: PLoS Biol. 2019 Mar 1;17(3):e3000161. doi: 10.1371/journal.pbio.3000161 (PMC6415873; doi:10.1371/journal.pbio.3000161)
Supplement: S3 Fig — Protocols are in [15,17] and references therein. The spurious band in Adora2a genotyping can be eliminated by using a hot start protocol. (PDF) [file pbio.3000161.s003.pdf]

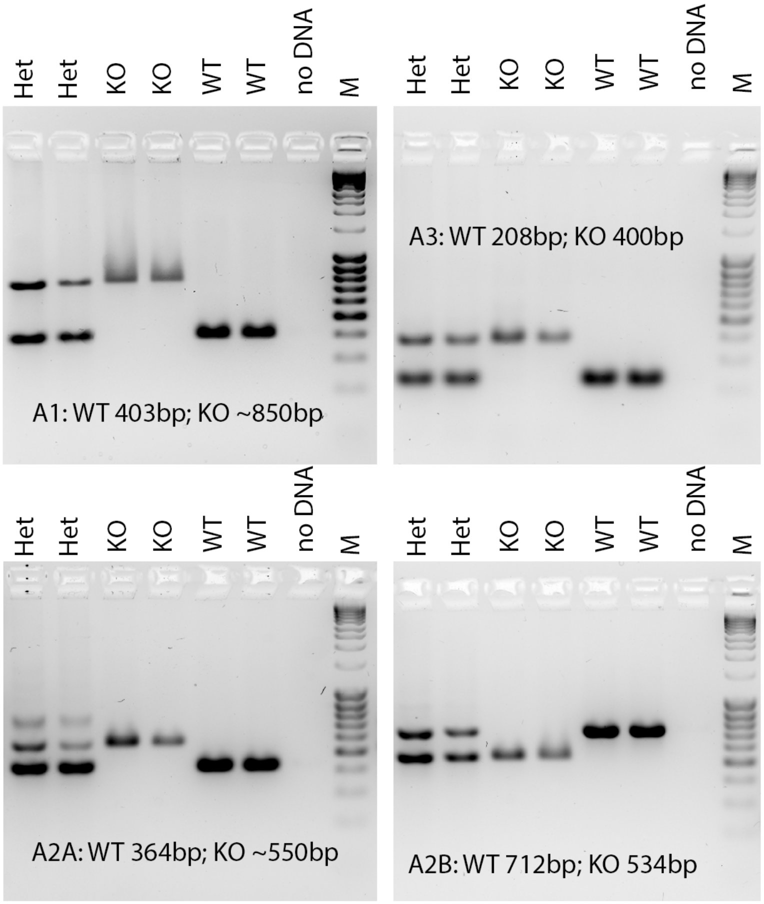

S3 Fig. Genotyping of *Adora1, Adora2a, Adora2b*, and *Adora3* alleles. Protocols are in [15, 17] and references therein. The spurious band in *Adora2a* genotyping can be eliminated by using a hot start protocol.

Primer	Primer sequence (5' to 3')	Direction
***Adora1* WT vs KO**		
x547	AGCTGGCTACCGCTACACAT	forward
x550	CCTGTTCCATGGCATCTCTT	reverse
x642 (Neo)	CATCGCCTTCTATCGCCTT	forward
***Adora2a* WT vs KO**		
x634	GGGCTCCTCGGTGTACAT	forward
x635	CCCACAGATCTAGCCTTA	reverse
x636 (PGK1)	CATTTGTCACGTCCTGCACGAC	reverse
***Adora2b* WT vs KO**		
x639	ACAGTAAAGACAGTGCCACCAG	forward
x640	AGACTTGTGTAACTCCAGGCAAG	reverse
x641 (Neo)	ATGGAAGGATTGGAGCTACG	forward
***Adora3* WT vs KO**		
x556	AGACAATGAAATAGACGGTGGTG	reverse
x557	ACTGGCCCATACACAACCTG	forward
x558 (Neo)	ATGGAAGGATTGGAGCTACG	reverse
