# Supplementary material for: Physiology and effects of nucleosides in mice lacking all four adenosine receptors
Source: PLoS Biol. 2019 Mar 1;17(3):e3000161. doi: 10.1371/journal.pbio.3000161 (PMC6415873; doi:10.1371/journal.pbio.3000161)
Supplement: S1 Table — QKO, quad knockout. (PDF) [file pbio.3000161.s015.pdf]

S1 Table. Hematology in Control and QKO mice.

| | **Control** | **QKO** | ***P*** |
|---|---|---|---|
| **White blood cell count (K/µL)** | 4.84 ±0.68 | 4.23 ±0.60 | 0.52 |
| **Red blood cell count (M/µL)** | 10.29 ±0.11 | 10.24 ±0.10 | 0.74 |
| **Hemoglobin (g/dL)** | 14.63 ±0.16 | 14.83 ±0.09 | 0.25 |
| **Hematocrit (%)** | 49.40 ±0.82 | 49.65 ±0.39 | 0.77 |
| **MCV (fL)** | 48.00 ±0.69 | 48.51 ±0.30 | 0.46 |
| **Platelets (K/µL)** | 864 ±53 | 1029 ±97 | 0.20 |
| **Polys (%)** | 24.0 ±4.8 | 13.3 ±1.0 | 0.021 |
| **Lymphocytes (%)** | 67.1 ±5.8 | 75.8 ±2.2 | 0.14 |
| **Monocytes (%)** | 1.90 ±0.17 | 1.05 ±0.11 | 0.0004 |
| **Eosinophils (%)** | 6.29 ±1.53 | 8.94 ±2.02 | 0.34 |
| **Basophils (%)** | 0.30 ±0.08 | 0.40 ±0.07 | 0.36 |

Male mice, 16-25 weeks old, mean ±SEM, n=8 control and n=11 QKO. *P* values are from unpaired t-Tests, without correction for multiple tests. MCV, mean corpuscular volume; polys, polymorphonuclear leukocytes.
