# Supplementary material for: Physiology and effects of nucleosides in mice lacking all four adenosine receptors
Source: PLoS Biol. 2019 Mar 1;17(3):e3000161. doi: 10.1371/journal.pbio.3000161 (PMC6415873; doi:10.1371/journal.pbio.3000161)
Supplement: S2 Table — QKO, quad knockout. (PDF) [file pbio.3000161.s016.pdf]

S2 Table. Serum chemistries in Control and QKO mice.

	Control	QKO	*P*
**Sodium (mmol/L)**	152.5 ±0.5	153.5 ±0.7	0.25
**Potassium (mmol/L)**	5.7 ±0.1	5.3 ±0.2	0.08
**Chloride (mmol/L)**	113.0 ±0.6	113.1 ±0.4	0.89
**Calcium (mmol/L)**	2.26 ±0.02	2.27 ±0.02	0.86
**Magnesium (mmol/L)**	1.09 ±0.03	1.12 ±0.03	0.50
**Phosphorus, inorganic (mg/dL)**	7.35 ±0.19	7.50 ±0.34	0.69
**Glucose (mg/dL)**	195.8 ±8.1	189.7 ±11.2	0.66
**Blood urea nitrogen (mg/dL)**	25.9 ±1.1	28.6 ±1.3	0.13
**Creatinine (mg/dL)**	0.12 ±0.01	0.12 ±0.01	0.85
**Uric Acid (mg/dL)**	1.79 ±0.23	1.51 ±0.23	0.40
**Albumin (g/dL)**	3.42 ±0.05	3.56 ±0.11	0.22
**Protein, total (g/dL)**	5.02 ±0.07	5.13 ±0.08	0.30
**Cholesterol (mg/dL)**	90.7 ±4.6	61.2 ±4.2	0.0001
**Triglycerides (mg/dL)**	95.1 ±13.9	81.8 ±8.7	0.45
**Alkaline phosphatase (U/L)**	60.3 ±5.0	111.3 ±12.5	0.0006
**Alanine amino transferase (U/L)**	30.7 ±1.6	34.0 ±2.5	0.27
**Aspartate amino transferase (U/L)**	102.0 ±4.9	102.3 ±5.5	0.97
**Amylase (U/L)**	2456 ±136	2129 ±114	0.09
**Creatine kinase, total (U/L)**	1306 ±132	936 ±88	0.038
**Lactate dehydrogenase (U/L)**	376 ±38	304 ±30	0.17
**Bilirubin, total (mg/dL)**	<0.2	<0.2	
**Bilirubin, direct (mg/dL)**	<0.2	<0.2	

Male and female mice, 30-37 weeks old, mean ±SEM, n=12 control and n=10 QKO. *P* values are from unpaired t-Tests, without correction for multiple tests.
