# Supplementary material for: Physiology and effects of nucleosides in mice lacking all four adenosine receptors
Source: PLoS Biol. 2019 Mar 1;17(3):e3000161. doi: 10.1371/journal.pbio.3000161 (PMC6415873; doi:10.1371/journal.pbio.3000161)
Supplement: S3 Table — Phenotype of older male mice. (PDF) [file pbio.3000161.s017.pdf]

S3 Table. Related to Table 1. Phenotype of older male mice.

	Control	QKO	*P*
**Weight (g)**	34.07 ±0.80	37.24 ±0.90	0.023
**Length (mm)**	88.29 ±0.84	90.00 ±0.38	0.07
**Inguinal WAT (g)**	0.402 ±0.057	0.616 ±0.065	0.029
**Epididymal WAT (g)**	1.10 ±0.21	1.37 ±0.11	0.25
**BAT (g)**	0.167 ±0.012	0.277 ±0.040	0.028
**Liver (g)**	1.29 ±0.04	1.52 ±0.05	0.006
**Quadriceps (g)**	0.418 ±0.023	0.413 ±0.005	0.80
**Spleen (g)**	0.095 ±0.005	0.093 ±0.006	0.80
**Kidney (g)**	0.541 ±0.015	0.537 ±0.017	0.89
**Heart (g)**	0.171 ±0.010	0.169 ±0.004	0.87
**Blood glucose (mg/dL)**	121.9 ±2.8	107.3 ±2.9	0.003
**Free fatty acids (mM)**	0.633 ±0.064	0.737 ±0.027	0.14
**Triglycerides (mg/dL)**	77.3 ±8.4	83.5 ±8.8	0.62
**Total cholesterol (mg/dL)**	128.7 ±8.0	98.0 ±8.9	0.025
**Insulin (ng/ml)**	0.93 ±0.10	1.92 ±0.38	0.034
**Leptin (ng/ml)**	7.8 ±1.5	20.4 ±4.9	0.038
**Adiponectin (µg/ml)**	13.45 ±2.44	14.48 ±0.71	0.67
**β-Hydroxybutyrate (mM)**	2.21 ±0.13	2.21 ±0.12	0.99
**Corticosterone (ng/ml)**	54.9 ±11.4	33.7 ±3.4	0.08
**Triiodothyronine (T3, ng/ml)**	0.581 ±0.057	0.820 ±0.045	0.006
**Thyroxine (T4, µg/dl)**	4.59 ±0.21	4.93 ±0.12	0.17

Male mice, 46 weeks old, mean ±SEM, n=7 controls and n=8 QKO. *P* values are from unpaired t-Tests, without correction for multiple tests. BAT, brown adipose tissue; WAT, white adipose tissue.
