# Supplementary material for: Physiology and effects of nucleosides in mice lacking all four adenosine receptors
Source: PLoS Biol. 2019 Mar 1;17(3):e3000161. doi: 10.1371/journal.pbio.3000161 (PMC6415873; doi:10.1371/journal.pbio.3000161)
Supplement: S4 Table — (PDF) [file pbio.3000161.s018.pdf]

S4 Table. Adipose tissue mRNA levels.

		N	Ucp1	Cidea	Cox8b
iWAT	WT	7	1.00 ±0.36	1.00 ±0.18	1.00 ±0.13
	QKO	5	8.29 ±5.84	2.18 ±0.87	1.09 ±0.27
	P		0.16	0.15	0.74
BAT	WT	7	337 ±56	33.7 ±3.2	9.66 ±0.62
	QKO	5	681 ±79	33.0 ±1.6	9.32 ±0.51
	P		0.0044	0.86	0.70

Data are mean ±SEM, are normalized to WT iWAT =1. *P* values are from unpaired t-Tests, WT vs QKO within tissue, without correction for multiple tests.
