# Supplementary material for: Physiology and effects of nucleosides in mice lacking all four adenosine receptors
Source: PLoS Biol. 2019 Mar 1;17(3):e3000161. doi: 10.1371/journal.pbio.3000161 (PMC6415873; doi:10.1371/journal.pbio.3000161)
Supplement: S5 Table — LPS, lipopolysaccharide; QKO, quad knockout; WT, wild-type. (PDF) [file pbio.3000161.s019.pdf]

S5 Table. Cytokine response to LPS in QKO and control (WT) mice.

**Group**	**WT vehicle**	**WT LPS**	**QKO vehicle**	**QKO LPS**	**WT LPS/vehicle**	**QKO LPS/vehicle**	**notes**	**2-way ANOVA *P* values**		
**N**	9	9	10	9				treatment	genotype	treatment x genotype
**CCL-11/Eotaxin**	1911 ±273	2259 ±313	1813 ±249	2279 ±359	1.18	1.26		0.18	0.90	0.84
**CCL2/MCP-1/JE**	817 ±472	34547 ±8039	187 ±0	17863 ±5632	42.29	95.69	A	<0.0001	0.08	0.10
**CCL3/MIP-1 alpha**	6.6 ±5.5	236.2 ±37.7	0.8 ±0.1	224.4 ±38.6	35.78	285.48	B	<0.0001	0.74	0.91
**CXCL-1/KC**	1797 ±1555	12708 ±1526	209 ±32	14174 ±60	7.07	67.85	B	<0.0001	0.95	0.16
**CXCL12/SDF-1 alpha**	1106 ±275	893 ±147	705 ±31	871 ±133	0.81	1.24		0.89	0.21	0.26
**Dkk-1**	6771 ±2326	3658 ±1431	4125 ±1615	3855 ±1667	0.54	0.93		0.35	0.50	0.43
**GM-CSF**	3.5 ±0.0	19.8 ±5.0	3.5 ±0.0	11.2 ±4.0	5.71	3.24	A	0.0004	0.17	0.17
**IFN-g**	7.0 ±1.8	25.4 ±6.4	8.2 ±3.8	13.7 ±4.0	3.61	1.66	A	0.0093	0.23	0.14
**IL-10**	17.0 ±7.6	69.3 ±19.7	9.3 ±0.0	108.0 ±57.6	4.08	11.59	A	0.0164	0.61	0.44
**IL-17E/IL-25**	40 ±12	264 ±53	26 ±5	203 ±39	6.53	7.74	A	<0.0001	0.26	0.48
**IL-27**	68 ±32	298 ±85	36 ±0	314 ±97	4.39	8.62	A	0.0004	0.90	0.72
**IL-6**	277 ±270	6512 ±1265	7 ±1	5285 ±1203	23.49	764.22	A,B	<0.0001	0.39	0.58
**MMP-12**	51 ±10	78 ±10	96 ±22	147 ±63	1.53	1.53		0.25	0.10	0.72
**TNF-alpha**	3.1 ±2.4	157.6 ±58.1	0.6 ±0.1	107.5 ±33.3	50.88	171.68	A	0.0003	0.43	0.47
**VEGF**	12.1 ±1.9	71.0 ±19.7	10.3 ±0.3	50.5 ±11.0	5.87	4.92		<0.0001	0.32	0.40
**CCL12/MCP-5**	106 ±24	984 ±336	83 ±7	1060 ±427	9.32	12.71		0.0013	0.92	0.85
**CCL-20/MIP-3 alpha**	29 ±1	527 ±242	124 ±77	948 ±513	18.48	7.62	A	0.0239	0.36	0.56
**CCL4/MIP-1 beta**	427 ±259	20606 ±7556	167 ±0	14721 ±5481	48.30	88.06	A	0.0006	0.50	0.54
**CXCL10/IP-10**	145 ±86	1764 ±540	59 ±0	1531 ±509	12.15	25.76	A	0.0002	0.66	0.84
**CXCL2/MIP-2**	62 ±58	2012 ±276	1.3 ±0.1	1808 ±249	32.35	1379.69	A,B	<0.0001	0.47	0.70
**EGF**	44 ±25	36 ±12	69 ±35	19 ±3	0.83	0.28		0.23	0.86	0.37
**G-CSF**	439 ±386	4641 ±586	62 ±14	4076 ±611	10.58	65.22	B	<0.0001	0.31	0.84
**ICAM-1**	19306 ±2627	27755 ±3115	19839 ±1996	24541 ±3073	1.44	1.24		0.0208	0.62	0.49
**IL-12 p70**	21 ±6	83 ±23	11 ±2	65 ±18	3.92	6.10		0.0004	0.33	0.79
**IL-17A**	13 ±4	52 ±31	13 ±4	26 ±10	4.17	2.01	A	0.11	0.42	0.41
**IL-2**	2.17 ±0.50	3.69 ±0.61	1.51 ±0.41	2.61 ±0.46	1.70	1.72		0.0128	0.09	0.67
**IL-4**	156 ±9	182 ±9	148 ±4	184 ±12	1.17	1.25		0.0009	0.72	0.56
**M-CSF**	1.9 ±1.0	102 ±31	0.7 ±0.2	67 ±25	52.44	94.38		0.0002	0.36	0.39
**MMP-8**	504746 ±201547	2060400 ±0	255378 ±23735	1954122 ±106278	4.08	7.65	B	<0.0001	0.12	0.53
**Renin**	10310 ±883	26329 ±6657	8655 ±389	21186 ±7630	2.55	2.45		0.0068	0.50	0.73
**TWEAK**	2.19 ±0.84	4.43 ±1.73	1.28 ±0.39	2.37 ±0.69	2.02	1.86	A	0.11	0.15	0.58
							**P <0.05 in:**	**24/31**	**0/31**	**0/31**

Mice were dosed with 250 µg/kg LPS and plasma obtained 2 h later.

A, levels in n≥3 control and n≥3 QKO vehicle-treated mice were ≤ lower limit of quantitation.

B, levels in n≥3 control and n≥3 QKO LPS-treated mice were ≥ upper limit of quantitation.
